# Song Is More Memorable Than Speech Prosody: Discrete Pitches Aid Auditory Working Memory

**DOI:** 10.3389/fpsyg.2020.586723

**Published:** 2020-12-10

**Authors:** Felix Haiduk, Cliodhna Quigley, W. Tecumseh Fitch

**Affiliations:** ^1^Department of Behavioral and Cognitive Biology, University of Vienna, Vienna, Austria; ^2^Vienna Cognitive Science Hub, University of Vienna, Vienna, Austria; ^3^Konrad Lorenz Institute of Ethology, University of Veterinary Medicine Vienna, Vienna, Austria

**Keywords:** language, music, memory, pitch, song, speech, auditory perception

## Abstract

Vocal music and spoken language both have important roles in human communication, but it is unclear why these two different modes of vocal communication exist. Although similar, speech and song differ in certain design features. One interesting difference is in the pitch intonation contour, which consists of discrete tones in song, vs. gliding intonation contours in speech. Here, we investigated whether vocal phrases consisting of discrete pitches (song-like) or gliding pitches (speech-like) are remembered better, conducting three studies implementing auditory same-different tasks at three levels of difficulty. We tested two hypotheses: that discrete pitch contours aid auditory memory, independent of musical experience (“song memory advantage hypothesis”), or that the higher everyday experience perceiving and producing speech make speech intonation easier to remember (“experience advantage hypothesis”). We used closely matched stimuli, controlling for rhythm and timbre, and we included a stimulus intermediate between song-like and speech-like pitch contours (with partially gliding and partially discrete pitches). We also assessed participants' musicality to evaluate experience-dependent effects. We found that song-like vocal phrases are remembered better than speech-like vocal phrases, and that intermediate vocal phrases evoked a similar advantage to song-like vocal phrases. Participants with more musical experience were better in remembering all three types of vocal phrases. The precise roles of absolute and relative pitch perception and the influence of top-down vs. bottom-up processing should be clarified in future studies. However, our results suggest that one potential reason for the emergence of discrete pitch–a feature that characterises music across cultures–might be that it enhances auditory memory.

## Introduction

Human communication is a fundamental behaviour defining how we see ourselves, particularly regarding our apparent uniqueness compared to other species. Two communicative systems are found in all human cultures, namely language and music, and both have key components executed in the vocal domain. Vocal music (song) and spoken language (speech) constitute two distinct but related modes of learned volitional vocalisations. The question of why humans engage in these two modes of vocalisations goes back to Darwin who proposed that they have a common origin (Darwin, 1871). They share the same vocal output system, which might be fundamental to the origin of both systems (see Levinson, 2013).

To clarify why humans engage in two modes of vocalisations–speech and song–a useful starting point is to break down these complex systems into components accessible for empirical study, in order to investigate the corresponding perceptual differences and their cognitive and behavioural consequences. Song and speech have multiple components in common. Both make use of the vocal apparatus (Lindblom and Sundberg, 2014) and share widely overlapping vocal production networks (Zarate, 2013; Pisanski et al., 2016). Both are complex auditory signals that rely on learned rules and are volitionally produced [for which the capacity of vocal learning is necessary; see Fitch and Jarvis (2013)]. Both consist of elements (for example notes or phonemes) that are generatively combined into sequences and group at different levels hierarchically, which exerts high demands on auditory memory. Finally, both modes of vocalisation are culturally transmitted and culturally variable (see e.g., Trehub, 2013; Mehr et al., 2019). However, song and speech are also perceptually clearly distinguishable, and not simply discriminated in arbitrary ways by different cultures (see Fritz et al., 2013). Aspects of the structure of the vocal spectrotemporal signal presumably influence this distinction. Furthermore, since these structural aspects are probably indicative of the underlying brain systems that shape the production and perception of song and speech (see e.g., Poeppel, 2011), signal structure offers a useful starting point to investigate their underlying cognitive and behavioural mechanisms.

Several structural aspects distinguishing music from language, termed “design features of music,” have been proposed by Fitch (2006). One of these design features concerns pitch, the perceptual component mostly based on the fundamental frequency (f0) of a sound. Pitch is an acoustic feature fundamental to both music (in song melody) and language (in speech prosody). Crucially, the variation of pitch over time, known as pitch trajectory or pitch contour, differs between the two modes, offering a well-defined perceptual component that contrasts between song and speech. In song, pitch contours typically consist of tones that are discrete in both time and frequency. The frequencies of these tones map onto culturally transmitted scales that themselves add structure to a musical phrase by relating pitches in particular ratios (intervals, see e.g., Krohn et al., 2007). Pitches and intervals provide the building blocks of melodies that can convey both affective and/or symbolic meaning to a culturally informed listener (see Seifert et al., 2013). In contrast, in speech, pitch contours are smoother and follow gliding up and down patterns. Speech intonation does not intentionally match specific pitches of a musical scale. This remains true in tonal languages, where either the direction of pitch shift (i.e., the contour) or the general relative level [high, low, medium pitch etc., see Bradley (2013)] is relevant, but not the specific absolute pitch. Nonetheless, speech intonation conveys considerable information in all languages, expressing emotional states, pragmatic and communicative intention, lexical meanings, etc (see Paulmann, 2016). Humans perceive and produce speech intonation constantly, starting in early childhood, and even prenatal exposure shapes neonates' vocal intonation production (Mampe et al., 2009). The interval relations between single tones in song have been suggested to utilise a fine-grained intonation perception system exclusive to music, while both song and speech would share a more coarse-grained perceptive system for the overall directional course of the intonation contour (Zatorre and Baum, 2012; see also Merrill, 2013). In summary, pitch supports a rich and overlapping body of communicative purposes in both song and speech, making it puzzling why these two distinct modes of utilising pitch would emerge in human vocalisations in the first place. A general auditory mechanism might become domain-specific by being utilised differently based on the nature of the input signal (phenotypic plasticity; Fritz et al., 2013). In turn a perceptual system that evolved for some other purpose might be exploited by a given signal structure, yielding a certain behavioural outcome (exaptation, Lloyd and Gould, 2017). It is also possible that a signal structure like discreteness of pitch in song is simply an epiphenomenon, without any behavioural consequences, but emerges due to sensory biases (see e.g., ten Cate and Rowe, 2007). Relating signal structure to cognitive consequences can therefore be informative about the underlying cognitive system, especially if it concerns a fundamental design feature that separates one signal type from others. Thus, it is worthwhile to investigate whether use of discrete vs. gliding pitches has functional consequences in auditory cognition.

Music and language both require auditory memory since both types of signals have variable content, and extend over time. Learned vocal phrases are important for song and spoken conversation. However, since music is not linguistically propositional (Fritz et al., 2013) it seems likely that auditory memory for song centres on spectrotemporal features like pitch. As for many studies comparing music and language, results concerning auditory working memory are mixed (see Schulze et al., 2018, for an overview). It has been suggested that remembering song and speech utilises two distinct systems of subvocal rehearsal: a phonological loop for verbal stimuli, and a tonal loop for musical sounds. Evidence for such a distinction comes from patient studies: Tillmann et al. (2015) reviewed pitch memory-deficits in congenital amusia (CA), concluding that there is a distinction between verbal and pitch-related memory systems, with only the latter being impaired in CA patients. However, CA patients do have difficulties in prosodic pitch perception as well (Tillmann et al., 2015), and it is unclear whether pitch-related memory is clearly divided into music-specific and speech-specific networks. Moreover, studies using intervening distractor sounds in a memory task found that pitch similarity of target (tones or words) and distractor sounds (words or tones) influences performance, suggesting an overlap in pitch-related working memory for song and speech prosody (Semal et al., 1996; Ueda, 2004). There is also evidence from fMRI studies that neural populations active in perception are recruited for subvocal rehearsal for working memory (Hickok et al., 2003), for both speech and melodies (see Buchsbaum, 2016). Hickok et al. (2003) also compared working memory maintenance of non-sense speech and piano music in these regions and found nearly identical time courses of activation. This might suggest substantial mechanistic overlap, and thus no substantial difference between memorability of sung and spoken stimuli.

However, a study by Verhoef et al. (2011) suggested that under demands for memory in a transmission task, random intonation contours morph into distinct elements that are then utilised in a combinatorial way. Using a slide whistle in an iterated learning task (where stimuli played by one participant were passed to another to imitate, and so on in a chain for many participants), pitch contours gradually appeared that were distinct and easy to memorise. However, no distinct scale tones emerged. Reasons might lie in the use of a non-vocal instrument, the structure of the initial contours in the iterative chains, or simply because there is no memory advantage for pitch contours consisting of discrete pitches over gliding intonation. Thus, it is not clear whether the pitch trajectory–discrete or gliding–by itself has an effect on memory for vocal phrases. This is the issue we aimed to address in the current studies.

We conducted three studies to test whether identical vocal phrases can be remembered better when consisting of discrete (song-like) or gliding (speech-like) pitches. We presented these stimuli in a same-different paradigm using a two-alternative forced-choice task, with an auditory distractor stimulus interposed between the target and test phases. We took care to minimise cultural biases, and we used various types of distractor sounds for each study, to vary task difficulty and to interfere with subvocal rehearsal. We also quantified musical experience in order to investigate its effects on auditory memory, since studies comparing musicians and non-musicians in terms of auditory memory often reveal advantages for musicians (see Schulze et al., 2018).

The existing literature reviewed above suggests several hypotheses and predictions. We first hypothesised that discrete pitches are a spectrotemporal property that enhances auditory memory (“song memory advantage”). This hypothesis predicts that vocal phrases consisting of discrete pitches should be remembered better than vocal phrases consisting of gliding pitches. Such a result would indicate that a fundamental and widespread acoustic feature of music has clear cognitive effects beyond surface-level perception. An alternative hypothesis is that gliding speech pitch might be remembered better, because humans perceive and produce speech prosody with high abundance from early childhood (“experience advantage”). The high familiarity of speech prosody might therefore make the gliding pitches of speech the more salient, and thus better-remembered, type of signal. Finally, based on previous literature we hypothesised that musical training might enhance memory effects, but we had no strong prediction about whether this advantage would apply either across stimulus classes, or specifically only for songlike stimuli with discrete pitch. Either way, the results should provide insight into the memory systems underlying these two systems.

## Study 1

### Materials and Methods

#### Stimuli

**Figure 1:**
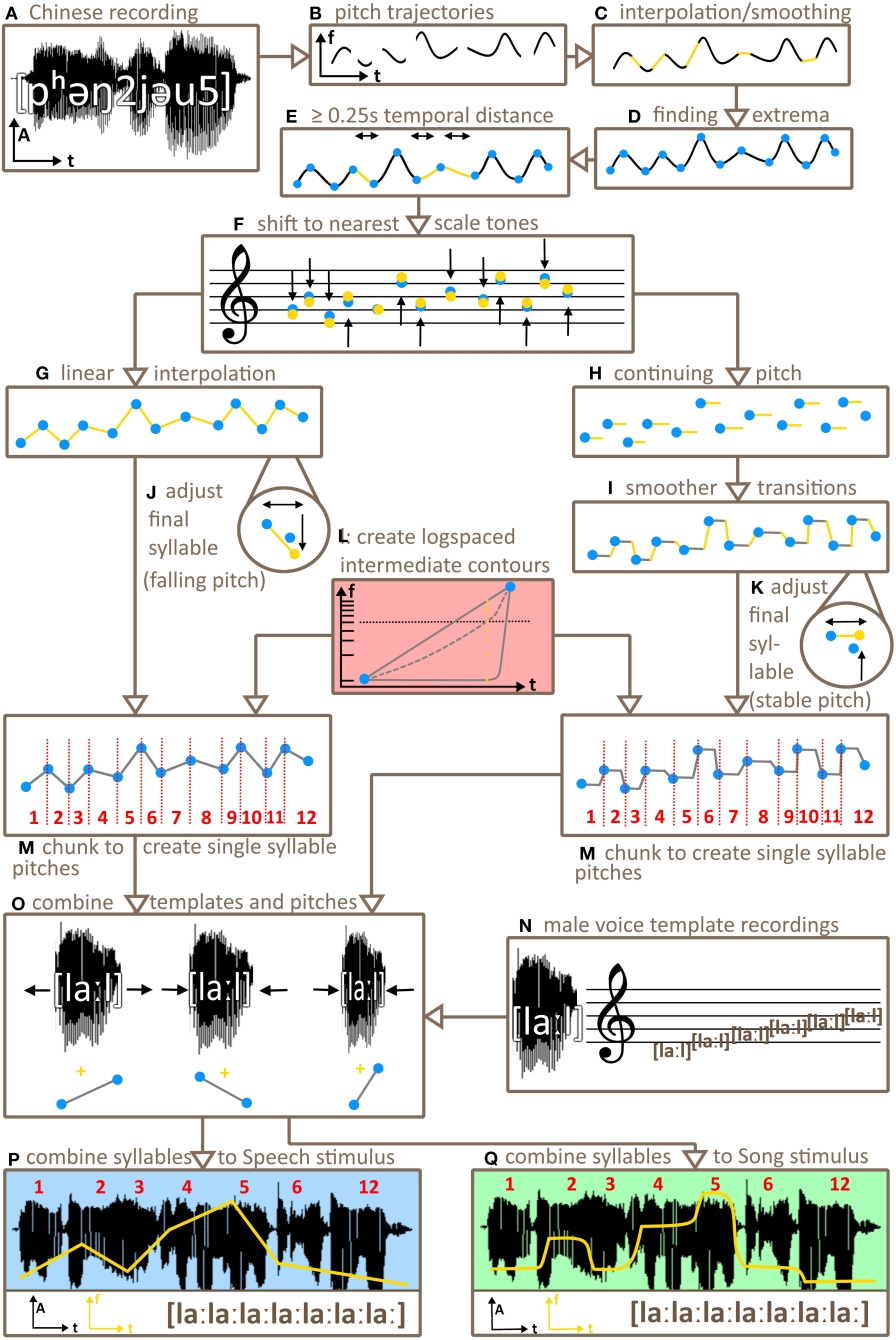
Schema of stimulus creation. From recordings of Mandarin Chinese phrases spoken by a male **(A)** pitch trajectories were extracted **(B)**, interpolated and smoothed **(C)**. After finding the local frequency extrema (minima/maxima) of the trajectories **(D)**, new pitch contours were derived by shifting the temporal distance between these extrema to create intervals of at least 0.25 s **(E)** and by subsequently shifting the extrema in frequency to the nearest Bohlen-Pierce or diatonic scale tones **(F)**. The type of interpolation between extrema was determined by stimulus category. For speech stimuli, Praat interpolated linearly between extrema **(G)**. For Song stimuli, pitch values at extrema were continued **(H)**, including a smooth transition right before the next minimum/maximum to avoid unnaturally abrupt pitch changes **(I)**. Pitch values for last syllables of each contour were adjusted to be 0.3s in duration, with falling pitch for Speech stimuli **(J)** and stable pitch for Song stimuli **(K)**. Seven intermediate interpolation variants on a continuum between Song and Speech stimuli were created **(L)**. Chunking pitch contours of all variants (Song, Speech, intermediates) using extrema as borders resulted in single pitch contour chunks **(M)**. One-syllable template sounds, recorded by a male on the syllable [la:l] at all Bohlen-Pierce scale tones **(N)** were selected to match the initial pitch of a pitch contour chunk. The respective chunk's duration and pitch contour were then carried over to the respective template sound **(O)** to derive stimulus syllables. Finally, the first six (studies 1 and 3) or nine (study 2) stimulus syllables and the respective last syllable of each contour and variant were concatenated to generate the final stimuli **(P,Q)**.

Our stimuli were based on natural speech samples, from which the pitch trajectory was extracted (see Figure 1). In order to minimize cultural influences due to familiarity these were Mandarin speech samples, spoken by an adult male^1^. For the same reason pitch tones were adjusted to lie on an unfamiliar Bohlen-Pierce-Scale. From each of the pitch trajectories new pitch contours were derived to keep the sentence-level prosodic movement. Note that Mandarin Chinese shows both sentence-level intonation and syllable-level lexical tone. Our manipulation removed syllable-level but maintained sentence-level intonation. Sentence-level intonation supports both semantic and phonological processing in native speakers of Mandarin Chinese, but not in non-speakers (Tong et al., 2005). However, as non-speakers of Mandarin Chinese still show behavioural and neuronal effects of prosodic processing (Tong et al., 2005), using Mandarin sentence-level prosody seems a reasonable choice to minimize effects of cultural familiarity. The pitch contours derived from the Mandarin speech samples were the basis for stimuli of two variants: one variant consisting of discrete tones (as in a song melodic intonation) and another variant consisting of gliding tones (as in speech prosodic intonation). Additionally, seven intermediate variants were created of which one was used as a stimulus halfway between discrete and gliding variants. The reason for this was to examine to what extent potential effects are based on bottom-up processing of pitch as opposed to top-down categorical processing (hearing the stimuli “as song” or “as speech”). We hypothesised that an intermediate stimulus would neither be perceived as clear song nor as clear speech prosody. If the predicted effect that discrete pitch aids memory was mediated by a categorical top-down perception of a stimulus “as song” we would expect the intermediate stimulus to show very minor effects only. If gliding pitch was more memorable, we also would expect only minor effects for intermediate stimuli since they would not represent clear speech prosody. If however any effects were based on bottom-up pitch perception, we would expect intermediate stimuli to show medium effects with a magnitude between our Song and our Speech stimuli, since the pitch is partly gliding and partly discrete. Finally, all pitch contours were re-synthesised using a one-syllable constant-pitch recording of [la:l] of a male singer. Final stimuli consisted of seven syllables for study 1 and study 3 and ten syllables for study 2.

The software package Praat (Version 6.0.36, Boersma and Weenink, 2017) was used to create the stimuli (see Figure 1). In a first step 200 pitch trajectories were isolated from 200 mandarin phrases, spoken by a male [function “To Pitch (ac),” for details on the parameters: see Supplementary Material].

Pitch trajectories were then smoothed using the function “pitchsmoothing” from Praat Vocal Toolkit (80%; Corretge, 2012) and unvoiced gaps were linearly interpolated using the praat standard function “interpolate.” Frequency values at local extrema (minima/maxima) of the smoothed pitch trajectory, along with the onset and offset frequencies of the whole pitch trajectory, were used as markers for deriving discrete pitches. These extrema were shifted in time, if necessary, to be at least 0.25 s distant from each other to later avoid unnaturally short tones/syllables. The frequency values at these extrema were shifted to the nearest Bohlen-Pierce scale tone, again to avoid any culture-specific biases. For a second variant frequency values at extrema were shifted to the nearest tone of the familiar western diatonic scale including both major and minor thirds. This resulted in new pitch contours, each in both a Bohlen-Pierce and a diatonic variant, both variants being equally tempered. Both the Bohlen-Pierce scale and the diatonic scale were based on 80 Hz as lowest tonic frequency and comprised 14 possible tones (see Table 1).

**Table 1:** Possible scale tones used in stimulus creation (both equal tempered, in Hz).

**#**	**Bohlen-pierce scale tones**	**Diatonic scale tones**	**Difference in cents**
1	80	80	0
2	87.1	89.8	52.85
3	94.7	95.1	7.3
4	103.1	100.8	−39.06
5	112.2	106.8	−85.39
6	122.1	119.9	−31.48
7	132.8	134.5	22.02
8	144.5	151	76.18
9	157.3	160	29.46
10	171.2	179.6	82.93
11	186.3	190.3	36.78
12	202.7	201.6	−9.42
13	220.6	213.6	−55.83
14	240	239.7	−2.17

*Equal tempered Bohlen-Pierce tones are about 146 cents apart from each other (ratio of ^13^√3), while equal tempered chromatic tones of a diatonic scale differ by 100 cents (^12^√2). The diatonic scale contained both major and minor thirds*.

To derive the discrete variant of each contour (henceforth Song stimuli), pitch samples between two adjacent extrema were set to the respective Bohlen-Pierce/diatonic tone of the first of the two extrema. This way stable pitches emerged between every two extrema. To ensure naturalistic sounding stimuli with no abrupt changes of pitch (that would have led to a yodelling sound), transitions between these stable pitches were smoothed by transitioning to the next pitch from 1/8 of the duration between two extrema before the next extremum. To derive the gliding contours, pitch samples between extrema were linearly interpolated. We henceforth refer to these stimuli as Speech stimuli for simplicity, noting that we investigated speech intonation, not full-fledged speech. Extrema were used as cut-offs to chunk whole contours into syllable-length PitchTiers. These chunks were later combined with the one-syllable male voice recordings [la:l]. The last chunk of a Speech contour was altered such that it always had a falling pitch from the final minimum/maximum and a duration of 0.3 s, while the last chunk of Song contours was altered such that it always had a sustained pitch and a duration of 0.3 s. This mimicked phrase-final lengthening that occurs in both natural speech and song (Arnold and Jusczyk, 2002). If the last minimum/maximum was near 80 Hz this led to some pitch samples below 80 Hz for the last syllables of some Speech stimuli (see Supplementary Table 1 for details).

To create stimulus variants between Speech and Song, we synthesised a continuum of seven intermediate stimuli between Song and Speech variants of each contour. To this end, frequency values (in Hz) of a stimulus' Song and the Speech variant at each given timepoint were converted to semitone values (“semitones re 1 Hz” in Praat). This way the difference between both values was converted from logspace (Hz) to linear space (semitones). This difference was then logarithmically divided into seven steps, resulting in seven values for the stimulus variants between Song and Speech. The logspacing was such that steps closer to Song were smaller than steps closer to Speech. Stimuli located perceptually halfway between Speech and Song would be categorised by a listener as Song (and not as Speech) 50% of times (which is called the Point of Subjective Equality, see Kingdom and Prins, 2016). To obtain an empirical estimate of this category boundary, we presented stimuli of all variants and contours to one participant (author FH) in a two-alternative forced-choice Speech/Song categorisation task, using a staircase method. We chose the variant closest to the Point of Subjective Equality as intermediate stimulus for study 1 (henceforth Intermediate). This was step 6, with Speech being step 1 and Song being step 9.

To obtain a naturalistic timbre, a male singer recorded sustained tones on the syllables [la:l] at each scale tone of the Bohlen-Pierce scale as well as the diatonic scale in the octave from 80 to 160 Hz (henceforth template syllables). Recording was done using a Zoom H4n recording device (16 bit, 44.1 kHz). In order to avoid template syllables at higher frequencies sounding more distant than template syllables at lower frequencies in the final stimuli and therefore disrupting the perception of a single speaker/singer (Bregman, 1994; Zahorik et al., 2005; Bregman et al., 2016), template syllables were not equalised in intensity. For each discrete Bohlen-Pierce /diatonic PitchTier chunk of each contour, the template syllable nearest in frequency to the respective pitch was adjusted in duration to match the duration of the respective PitchTier chunk, whereby the first and last 0.02 s of each template syllable remained unstretched in time in order to keep the liquid consonant [l] unchanged. The pitch contour was then combined with the spectral information of the template syllable and resynthesised to a syllable [la:l] following the current pitch contour. This procedure resulted in the final syllable sounds (henceforth syllables) for each contour and variant (i.e., Song, Speech and the seven Intermediates). The first six and the last syllable of each contour and variant were then concatenated with an overlap of 0.03 s, and the resulting stimulus was ramped using a trapezoid with 0.01 s rise and fall. Thus, the same syllable sequence with the same durations for each syllable was used for all variants of each contour (i.e., Song, Speech, and the Intermediate stimuli). The mean duration of syllables across all stimuli used was 0.360 s (SD = 0.168). The mean pitch acceleration rate of syllables across the speech prosody stimuli was 0.106 Hz/ms (SD = 0.081), thus much lower than for lexical tones in Mandarin Chinese (see Krishnan et al., 2010). Stimuli were scaled in intensity to 73 dB SPL (Praat: “Scale intensity”; i.e., the root-mean-square amplitude of the stimuli was changed to 73 dB above 0.00002 Pa). All stimuli were monophonic, 16 bit and with a sampling rate of 44.1 kHz.

For study 1 and 3, only stimuli with a duration between 2 and 3 s and with 7 syllables were used. For study 2, only stimuli between 3 and 4 s of duration and with 10 syllables were used. Note that syllable durations were ultimately based on the natural speech signals we used (Mandarin Chinese) and that therefore our stimuli were not isochronous or metrical in any sense.

Stimuli that were too similar to each other were excluded (evaluated aurally by author FH), resulting in 94 different stimulus contours per scale (diatonic/Bohlen-Pierce), each as Song, Speech and Intermediate variants.

To create the deviant stimuli for the same/different memory task, the pitch of one randomly chosen syllable (target syllable) of each contour, excluding the first and the last syllable, was shifted to another of the scale tones of the Bohlen-Pierce/diatonic scales specified above. The direction of the shift reversed the direction of pitch change between the target syllable and the syllable preceding it, such that a rising pitch interval between target syllable and preceding syllable became falling after the shift and vice versa. That way the overall pitch trajectory, that is the global up and down of the pitch contour, always changed. Exceptions were cases when the shift would have resulted in pitches of target syllables outside the Bohlen-Pierce/diatonic scales specified above. In such cases the pitch interval between two consecutive syllables changed in magnitude but not in direction (falling or rising). This was the case for 280 out of the 3,720 trials that made up the sample for study 1. The amount of pitch shift was random within the constraints just mentioned (see below in the statistical methods for details on the pitch deviation). The mean absolute pitch deviation was 3.8 semitones (SD = 5.0).

Distractor sounds matching the temporal structure of the tonal stimuli were constructed from pink noise bursts of random duration between 0.2 and 0.3 s in Praat (decreasing by 6 dB SPL per octave, F0 = 100 Hz) and concatenated with 0.01 s overlap, such that the total duration of the distractor was 2 s. Two hundred and eighty two different distractors were created to allow unique distractors to be used for each variant of each contour.

Distractor sounds and stimuli where presented such that in the same-different task participants would hear an original stimulus contour first, then a distractor sound and then either the original stimulus contour again (same) or the respective deviant stimulus contour (different).

#### Procedure

Thirty three participants (21 females, 12 males, age range 18 to 56 years, mean = 24.3 years) took part in the study. Throughout all three studies, participants were recruited via posters at the university venue and via Facebook and most of them were university students from the University of Vienna. All participants in the three studies were rewarded with 5 € per half hour of participation (approx. study duration was 60 min for all three studies). All participants in the three studies gave written informed consent to participate. All three studies were approved by the ethics committee of the University of Vienna (# 00361).

Participants were welcomed by the experimenter (author FH) and led to one of two adjacent testing rooms for human participants where they were seated in front of a computer. To avoid expectancy effects participants were given minimal, written instructions in German or English^2^. They were told simply that their task was to make decisions about sounds by clicking on circles on a screen. Words such as “song,” “speech,” “language,” “music” were not mentioned.

Python 2.7 was used to present stimuli (scripts based on the script-building program “Experimenter_GUI, version 0.1” by Pinker, 1997). Sounds were played via Sennheiser HD 201 headphones at about 73 dB SPL. The study was run on a Macbook Pro laptop (Retina display, 15-inch screen, Mid 2015). Except during the training phase, the experimenter left the testing room so that each participant executed the tasks alone.

The experiment consisted of four phases: a training phase for the same-different task (about 10 min duration), the same-different task itself (about 30 min), a test of spectral or holistic hearing (Schneider et al., 2005) and the Gold-MSI musicality questionnaire (Müllensiefen et al., 2014). We did not include the results from the test of spectral or holistic hearing as results were heavily skewed towards holistic hearing (holistic:mix:spectral hearing 16:15:2 in study 1) and therefore uninformative.

Instructions were provided in written form on the computer screen. Trials were self-paced so participants could take breaks between trials and after each part of the experiment.

In the initial training phase, participants were asked to judge whether a “sound example” (German: “Klangbeispiel”) changed or stayed the same and were instructed that their goal was to gain as many points as possible by answering correctly. They could start each trial by clicking on a small white filled circle in the middle of a grey screen and made decisions by clicking on one of two rectangular response boxes on the left and right side of the screen (side randomised between trials). One response box contained the word “anders” (or “different”), the other one “gleich” (or “same”). In order to minimise lapse errors due to random change of the sides where two boxes would appear, boxes were consistently outlined by rectangular lines of yellow (“same”) or blue (“different”). Participants were informed that their current points score would be shown on the screen after each trial. After reading these instructions, participants were allowed to ask clarification questions. No information concerning the task purpose, the nature of the stimuli or the type of change in difference stimuli was given at any point during the study.

In the training phase, both Song and Speech variants of ten different pitch contours randomly chosen from the pool of diatonic pitch contours (thus 20 stimuli) were presented in random order in the two-alternative forced choice design. Pitch contours used in the training phase were not used in the later test phase to avoid any memory transfer effects. Participants first heard one stimulus, followed by the distractor sound, and then either the stimulus again (same) or its deviant version (different). Thus, in cases when deviants occured they were always played after the original (unaltered) stimulus.

After clicking on one of the two response boxes, the participants received auditory and visual feedback. Correct choices were rewarded by displaying a gain of five points and a short bell-like sound. Incorrect choices were punished by the screen turning red for 500 ms along with playing an aversive sound and by displaying a loss of five points. Failed trials were repeated until successful. The current number of points (which could only be positive) was also displayed after each trial.

During this initial training phase, the experimenter stayed in the room to allow the participants to ask questions about the task.

The second phase of the same-different task was the test phase and involved 120 forced-choice trials. Participants first received instructions written on the computer screen. The instructions given were the same as in the training phase, except that participants were told that the following task would consist of three blocks with an approximate duration of 10 min each and the possibility to take a break of minimum 30 s between blocks. Additionally, participants were informed that points they gained would now only be displayed after each block (no feedback after trials). After the instruction, participants were allowed to ask clarification questions.

After the instructions the experimenter left the room until the participant was finished.

The same setup was used as in the training phase. This time, 40 different pitch contour pairs, randomly drawn from the pool of Bohlen-Pierce stimuli individually for each participant, were presented in each the Speech variant, the Song variant, and the Intermediate variant (in sum 120 trials). Half of the stimuli were difference stimuli (i.e., included a pitch change on one syllable during the second presentation of the pitch contour after the distractor noise) and half of the stimuli were without change.

Presentation order of the stimuli was random. In total 120 trials were presented. After each block of 40 trials, the run was interrupted for 30 s and written instructions appeared on the screen, asking the participant to take a short break and continue by clicking the white circle. To ensure a high level of motivation throughout the task an arbitrary current point score was displayed as well, showing 135 points after the first block and 260 points after the second block.

Participants were also instructed to self-assess their current concentration abilities after each block on a 7-point Likert scale. This was done to assess the reliability of the results for each block, whereby our a-priori criterion was to drop blocks for which concentration was below 3. During the test phase, no feedback was given after each trial and no trials were repeated. After the participants were finished, the experimenter re-entered the room.

Part three of the study consisted of the test on holistic vs. spectral hearing from Schneider et al. (2005), which we did not use in the analysis because of biased results.

Part four of the study consisted of the Gold-MSI test to assess musicality of the participants (Müllensiefen et al., 2014), and a post-experimental questionnaire asking about strategies the participants used, any speculations about the purpose of the study and the languages they spoke (including level of proficiency).

Two speakers of Mandarin Chinese were excluded to avoid any familiarity confounds due to the original contours being Chinese.

Afterwards, participants gave post-experimental consent for using their data, were thanked, paid, and accompanied to the exit. The total duration of part 1 of the experiment ranged from 50 to 90 min (two participants needed more than 60 min due to the self-paced procedure).

#### Statistical Methods

Our goal was to quantify whether vocal contours are remembered better when consisting of discrete rather than gliding intonation contour. Our memory task required a binary response (original and target contour judged to be different or same), given two possible states of the target signal (original and target contour deviate or do not deviate). Our task therefore can be analysed in the framework of signal detection theory (see Kingdom and Prins, 2016, for an overview).

To this end, we used a logistic GLMM with logit link function. The response variable was the response given by the participants (“Response Given”). Fixed effects entered were the response that would have been correct (“Stimulus State”) and the variant of the contour (“Variant” Song, Speech, Intermediate) along with their interaction. We z-transformed the musicality score of the Gold-MSI in order to obtain model estimates for participants of average musicality (“z.Musicality”) and included this as a fixed effect as well. As random slopes we entered “Stimulus State,” “Variant” and “z.Musicality” within “Contour” (the 94 different stimulus pitch trajectories created) and “Stimulus State” and “Variant” within “Participant.” Interactions of random intercepts and random slopes were not included. We also did not include semitone deviation as a predictor in the model since it did not differ significantly between Variants (Median_Speech_: 6.67; Median_Intermediate_: 8.6; Median_Song_: 7.88; Kruskal Wallis Test: H = 0.51, df = 2, *p* = 0.78; note that the semitone differences were derived in Praat as real numbers, not integers). We did not include the results of the test of spectral vs. holistic hearing as its results were heavily skewed towards holistic hearing (only 1 out of 31 participants in the analysis sample scored as spectral hearer). Individual participants (“Participant”) and stimulus contours (“Contour”) were included as random intercepts. Dummy coding was done by a custom-built function. Optimizer “bobyqa” with maximum 100,000 function iterations was used to assess the maximum likelihood.

All statistical analyses were done in R (R Core Team, 2019). We fitted the model (and all following models) using the function glmer (R package “lme4”; version 1.1-21; Bates et al., 2015).

Collinearity was assessed by fitting a linear model without random effects and interactions between fixed effects and applying the function “vif” (R package “car”; version 3.0-2; Fox et al., 2011). The function revealed no collinearity issues.

Overdispersion was tested using a custom-built function. The model was not overdispersed, and in fact appeared to be slightly underdispersed (χ^2^ = 2831.574, df = 3699, *p* = 1, dispersion parameter = 0.77), so no further action was required.

To assess how robust the model estimates would be given changes in the predictors we assessed model stability using a custom-built function. This function excludes participants one at a time from the data. Model estimates of the reduced and the full datasets are then compared (please see Nieuwenhuis et al., 2012 for a comparable approach). The model turned out to be stable (for ranges of parameter estimates of fixed effects please see Supplementary Table 2 and Supplementary Figure 1).

In order to test the effect of all predictors as a whole we compared the full model with a null model lacking the predictors of interest, i.e., including only the random factors. Models were compared using a likelihood ratio test, applying the function “anova.glm” (argument test set to “Chisq”; Dobson, 2002; Forstmeier and Schielzeth, 2011).

To test the effect of single predictors we used likelihood ratio tests, comparing the full model with a model reduced by one of the effects of interest (R function “drop1” with argument “test” set to “Chisq”). We first assessed the effect of the three-way interaction “Stimulus State:Variant:z.Musicality.” To test the effect of all two-way interactions, including the interaction of interest “Stimulus State:Variant,” we fitted the model without the three-way interactions and executed likelihood ratio tests as above.

The sample size for this model was 31 individuals with 120 observations each, thus a total of 3,720 observations.

We derived 0.95 confidence intervals using the function “bootMer” (package “lme4”) with 1,000 parametric bootstraps.

After applying the analyses mentioned above, we additionally did a repeated-measures Anova (R function “aov_ez,” from package “afex,” version 0.26-0; Singmann et al., 2020) on classically calculated d-primes for informative purposes (correction for hit rates and false alarm rates of 0 and 1: +− 1/(2N). A hit was defined as correctly detecting a difference (deviation), a false alarm accordingly was a response as “different” while there was no change in the stimulus. Corrections had to be done for 17 of 93 cells for this analysis). Note that this approach goes along with a loss of information in the data and is therefore not the ideal way of dealing with a data set with binary responses. We included “Variant” as repeated measures factor.

### Results

#### Post-experiment Questionnaire

Twelve participants reported that they had attempted to repeat the stimuli silently or by humming to remember them, some of these participants additionally tapped their fingers. One participant reported to have visualised the contours, one used only finger tapping as strategy. Four participants guessed that the study was about memory. This suggested that the distractor sound we used was not distracting enough which was one reason to replicate the study under more difficult conditions.

#### Statistical Results

**Figure 2:**
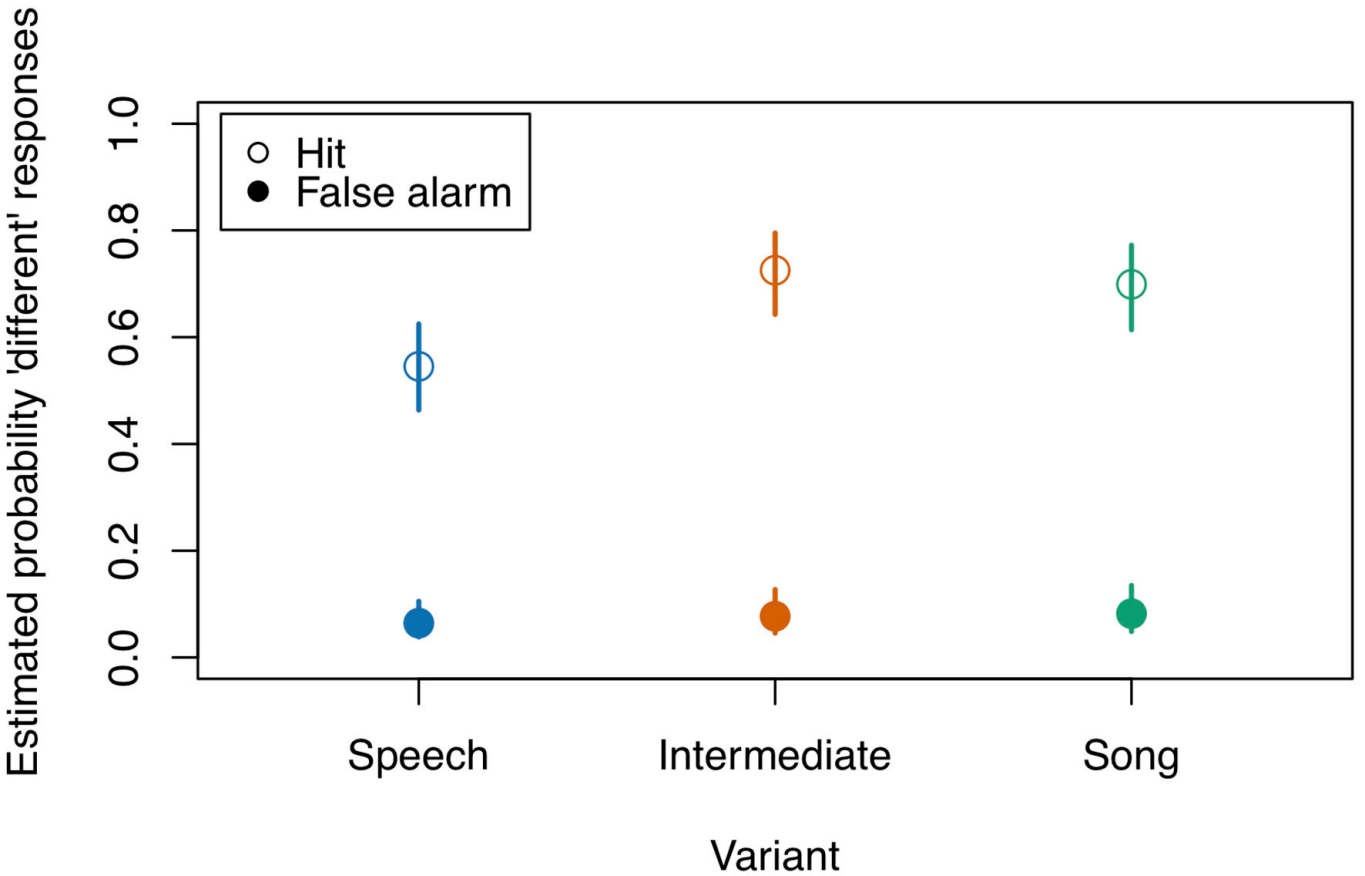
Model estimates for the probability of detecting a deviant in study 1 (see also Table 3). Empty circles: Hits, filled circles: False Alarms. Bars indicate 0.95 confidence intervals. *N* = 31.

After fitting the full model (model coefficients see Table 2) we first assessed whether it differed significantly from the null model (comprising only the random factors). We found that all predictors of interest as a whole explained the data significantly better than the null model (χ^2^ = 117.436, df = 11, *p* < 0.001). Figure 2 and Table 3 show the probability estimates of the full model.

**Table 2:** Estimates of model coefficients (log odds), standard errors (SE), lower (CI_lower_) and upper (CI_upper_) 0.95 confidence intervals, z statistics (*z*-value) and associated *p*-values of the model fitted for study 1.

**Term**	**log odds**	**SE**	**CI_**lower**_**	**CI_**upper**_**	***z***	***p***
Intercept	−2.678	0.275	−3.229	−2.166	−9.735	
Stimulus state diff	2.860	0.254	2.391	3.378	11.281	0.000
Intermediate	0.197	0.209	−0.240	0.643	0.941	0.347
Song	0.264	0.210	−0.175	0.692	1.257	0.209
z.Musicality	0.295	0.247	−0.217	0.768	1.191	0.234
Stimulus state diff: Intermediate	0.593	0.239	0.127	1.079	2.477	0.013
Stimulus State diff: Song	0.397	0.236	−0.084	0.882	1.680	0.093
Stimulus state diff:z.Musicality	0.016	0.234	−0.442	0.488	0.070	0.945
Intermediate:z.Musicality	−0.309	0.173	−0.683	0.084	−1.787	0.074
Song:z.Musicality	−0.086	0.174	−0.481	0.324	−0.490	0.624
Stimulus state diff: Intermediate:z.Musicality	0.616	0.225	0.142	1.119	2.742	0.006
Stimulus state diff:Song:z.Musicality	−0.010	0.223	−0.468	0.476	−0.044	0.965

*The model estimated detection of a difference between original sound and target sound (reference level: Stimulus State same) as a function of Stimulus Variant (Speech, Song, Intermediate. Reference level: Speech) and Musicality as assessed by the GoldMSI questionnaire and z-transformed (that way the estimates refer to an individual with average musicality)*.

**Table 3:** Probability estimates and 0.95 confidence intervals for Hits and False Alarms as depicted in Figure 2.

	**Speech**			**Intermediate**			**Song**		
**Stimulus state**	**Estimate**	**CI_**lower**_**	**CI_**upper**_**	**Estimate**	**CI_**lower**_**	**CI_**upper**_**	**Estimate**	**CI_**lower**_**	**CI_**upper**_**
Diff	0.545	0.463	0.625	0.725	0.643	0.795	0.699	0.614	0.772
Same	0.064	0.039	0.105	0.077	0.046	0.127	0.082	0.049	0.135

*Derived from the R-package “effects”, version 4.1-0 (Fox et al., 2019)*.

**Figure 3:**
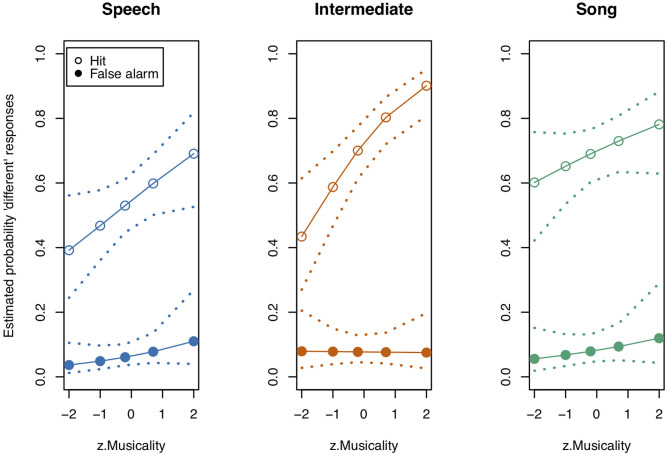
Model estimates for Musicality on the probability of detecting a deviant in study 1. Musicality had been assessed with the Gold-MSI and z-transformed for analysis. Empty circles: Hits, filled circles: False Alarms. Dotted lines represent 0.95 confidence intervals. Musicality showed the strongest impact for Intermediate stimuli. *N* = 31.

We next tested the three-way interaction “Stimulus State:Variant:z.Musicality,” which was significant (LRT = 9.686, AIC = 3,556.861, df = 2, *p* = 0.008, Nagelkerke's *R*^2^ = 0.046). Musicality enhanced memorising much more for Intermediate stimuli than for Speech or Song stimuli. Note that therefore the interpretation of the two-way interactions was not straightforward anymore (see Figure 3 and Table 4 for detailed results on the two-way interactions). We found that the interaction “Stimulus State:Variant” was significant, that is, participants could memorise both Song and Intermediate stimuli better than Speech stimuli (see also Figure 2), with the magnitude depending on their Musicality score.

**Table 4:** Degrees of freedom (Df), Akaike Information Criteria (AIC), Likelihood Ratio test statistic (LRT), associated *p*-value [Pr(Chi)] and Nagelkerke's R^2^, based on likelihood ratio tests for two-way interactions of the model fitted in study 1.

**Term**	**Df**	**AIC**	**LRT**	**Pr(Chi)**	**Nagelkerke's *R*^**2**^**
Stimulus state:Variant	2	3559.364	6.503	0.039	0.043
Stimulus State:z.Musicality	1	3556.025	1.164	0.281	0.035
Variant:z.Musicality	2	3554.465	1.604	0.448	0.017

For the Anova, we assessed the assumption of normality of residuals using a qqplot and histogram of the residuals, which showed no obvious deviation from normality. The assumption of sphericity had not been violated (Mauchly's Test of Sphericity: W = 0.984, *p* = 0.793).

We obtained a trend for a difference between Variants [*F*_(2, 60)_ = 2.5, *p* = 0.09, generalised η^2^ = 0.03]. For an overview of the distribution of d-primes as function of Stimulus Variant please see Supplementary Figure 2, for the raw hit and false alarm rates please see Supplementary Table 3.

## Study 2

Study 1 showed the predicted effect of discrete pitch on memory. To test how robust and far-reaching the effect would be, and in particular whether it would be present in a much more difficult task, we replicated study 1 with more challenging settings. To increase difficulty, we decreased the magnitude of deviation in the target stimuli, increased the number of syllables in each stimulus and changed the distractor sound. Moreover, given that we found that Intermediate stimuli had similar effects to Song stimuli, we replicated study 1 with an Intermediate stimulus closer to Speech stimuli. We hypothesised that the effects of Intermediate stimuli would then be between those for Song and Speech stimuli.

### Materials and Methods

#### Stimuli

The same procedure for stimulus creation was used as for study 1 except that now 10 syllables were used per stimulus contour and the duration limits changed to 2–4 s (as opposed to 7 syllables and a duration between 2 and 3 s in study 1). To this end the first nine syllables and the last syllable of each contour were concatenated. Also, we reduced the pitch shift of the target syllable from the template stimulus in comparison to study 1 (mean = 1.1 semitones [absolute], SD = 1.4). Two variants were created of each deviant: one where the pitch change went in the same direction between target syllable and its predecessor as before the shift, and one where the change was in the opposite direction. Moreover, the distractor sound was changed such that white noise was band-pass filtered (Hann) with a random value between 50 and 300 Hz for each burst as the lower cut-off and 10 times this value as the upper cut-off (smoothing: 10 Hz). A unique distractor sound was assigned to each variant of each contour. Since in study 1 both Song and Intermediate stimuli showed very similar effects, we decided to use a stimulus variant more similar to Speech. Of the seven intermediate variants that made a continuum between Speech and Song we used the variant one step closer to Speech (i.e., step 5 as compared to step 6 in study 1).

#### Procedure

Forty participants (35 females, 5 males, mean age = 21.5 years, range 19 to 54 years) took part. The same experimenter as in study 1 ran all participants.

The procedure was exactly the same as for study 1, except that study 2 took place in a sound proof chamber. The total duration of part 2 of the experiment ranged from 50 to 105 min. Three participants needed more than 60 min due to the self-paced procedure, one of which took 105 min to complete the experiment. To avoid that speakers of tonal languages would interpret stimuli differently on the basis of intonation than other participants, we excluded speakers of tonal languages. Therefore, one speaker of Mandarin Chinese and one speaker of Vietnamese were excluded from the sample for statistical analysis.

#### Statistical Methods

**Figure 4:**
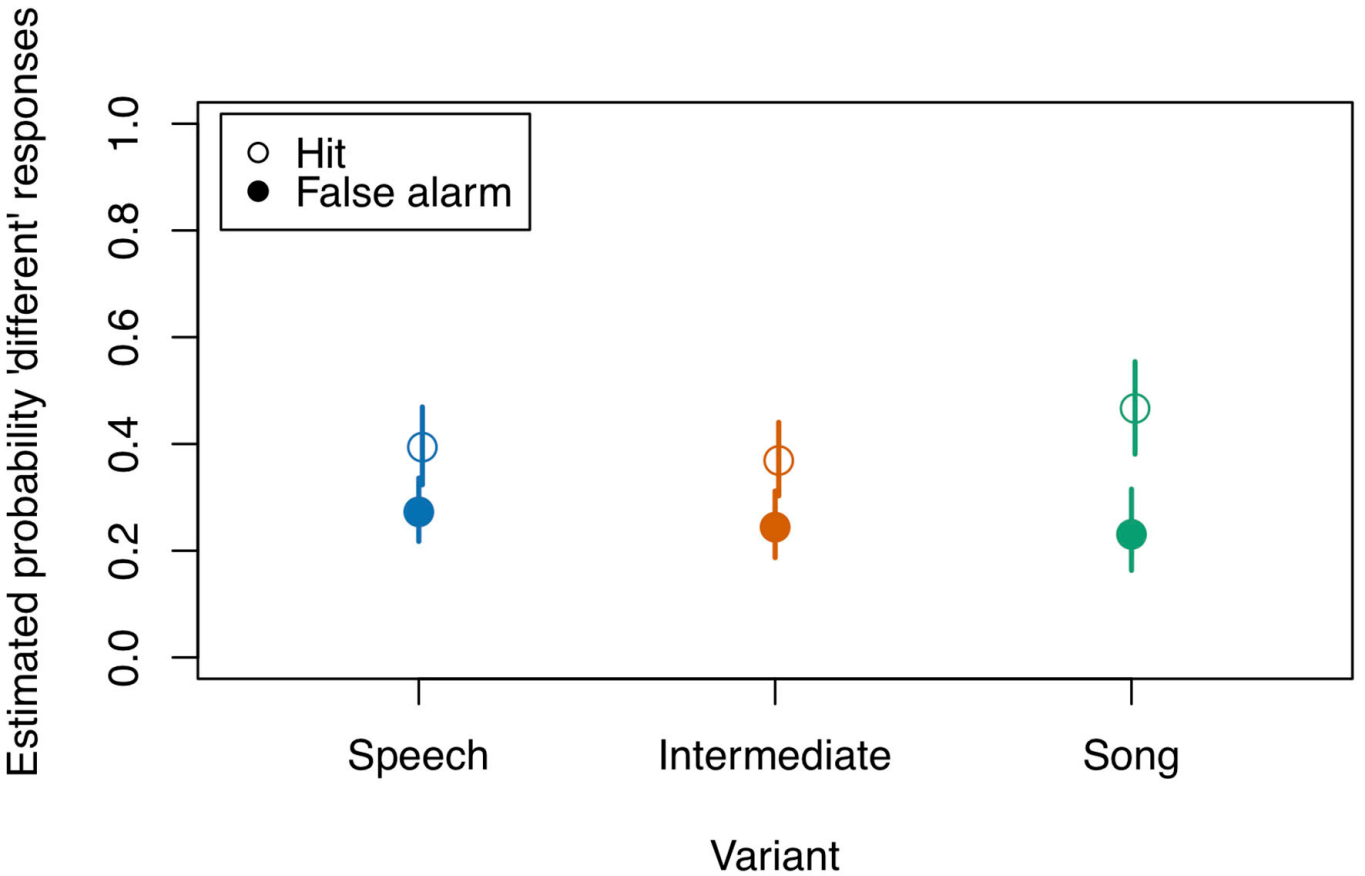
Model estimates for the probability of detecting a deviant in study 2. Empty circles: Hits, filled circles: False Alarms. Bars indicate 0.95 confidence intervals. Note that the probability of detection is overall very low. *N* = 38.

The data showed very different effects in comparison to the data of study 1 (see Figure 4). Calculating d-primes the traditional way (see Supplementary Figures 2–4) showed much lower sensitivity in study 2 (mean d-prime = 0.48, var = 0.26) compared to study 1 (mean = 1.65, var = 0.49) (study 3 showed intermediate values: mean = 1.08, var = 0.65). We therefore concluded that this task was quite difficult to solve for the participants.

We fitted a logistic GLMM with logit link function with the same parameters as in study 1. Again, interactions of random intercepts and random slopes were not included. We attempted to limit semitone deviation in this study to 1 or 2 semitones and therefore did not include this factor in the model. Although the limitation worked on average (mean = 1.1 semitones [absolute], SD = 1.4), we found in a *post-hoc* analysis of stimuli that the deviation surpassed this limit for some contours, yielding a significant difference between stimulus variants in terms of deviation (Median_Speech_: 2.27; Median_Intermediate_: 2.29; Median_Song_: 1.61; Kruskal Wallis Test: *H* = 29.68, df = 2, *p* < 0.001). However, including absolute semitone deviation as an additional random factor in the model yielded slightly different results, but did not change the interpretation from the original model, so we only report the original model here. Trials of blocks for which participants indicated a concentration level below 3 (on a 7-point Likert scale with 1 being lowest concentration) were excluded from the data. This concerned three blocks in total, from two participants, excluding 120 out of 4,560 trials. Thus, 4,440 observations from 38 individuals remained as sample size for the model.

Model stability was confirmed using a custom-built function (for ranges of parameter estimates of fixed effects please see Supplementary Table 4 and Supplementary Figure 5).

Collinearity was assessed as for study 1 and no collinearity issues were found.

Testing overdispersion revealed that the model was slightly underdispersed (χ^2^ = 3685.337, df = 4019, *p* = 0.99, dispersion parameter = 0.92), so no further action was required.

Full-null model-comparison and tests of fixed predictors were done using likelihood ratio tests as in study 1.

We derived 0.95-confidence intervals using the function “bootMer” (package “lme4”) with 1,000 parametric bootstraps.

After fitting this model, we additionally did a repeated-measures Anova on classically calculated d-primes for informative purposes with the same parameters as in study 1 (Corrections for hit rates and false alarm rates of 0 and 1 had to be done for 3 of 114 cells for this analysis). *Post-hoc* tests were calculated using the package “lsmeans,” version 2.30-0 (Lenth, 2016).

### Results

#### Post-experiment Questionnaire

Fourteen participants reported they attempted subvocal rehearsal, three of these participants also visualised the stimuli. Four additional participants reported visualising the stimuli. Two participants guessed that the study was about memory.

#### Statistical Results

**Figure 5:**
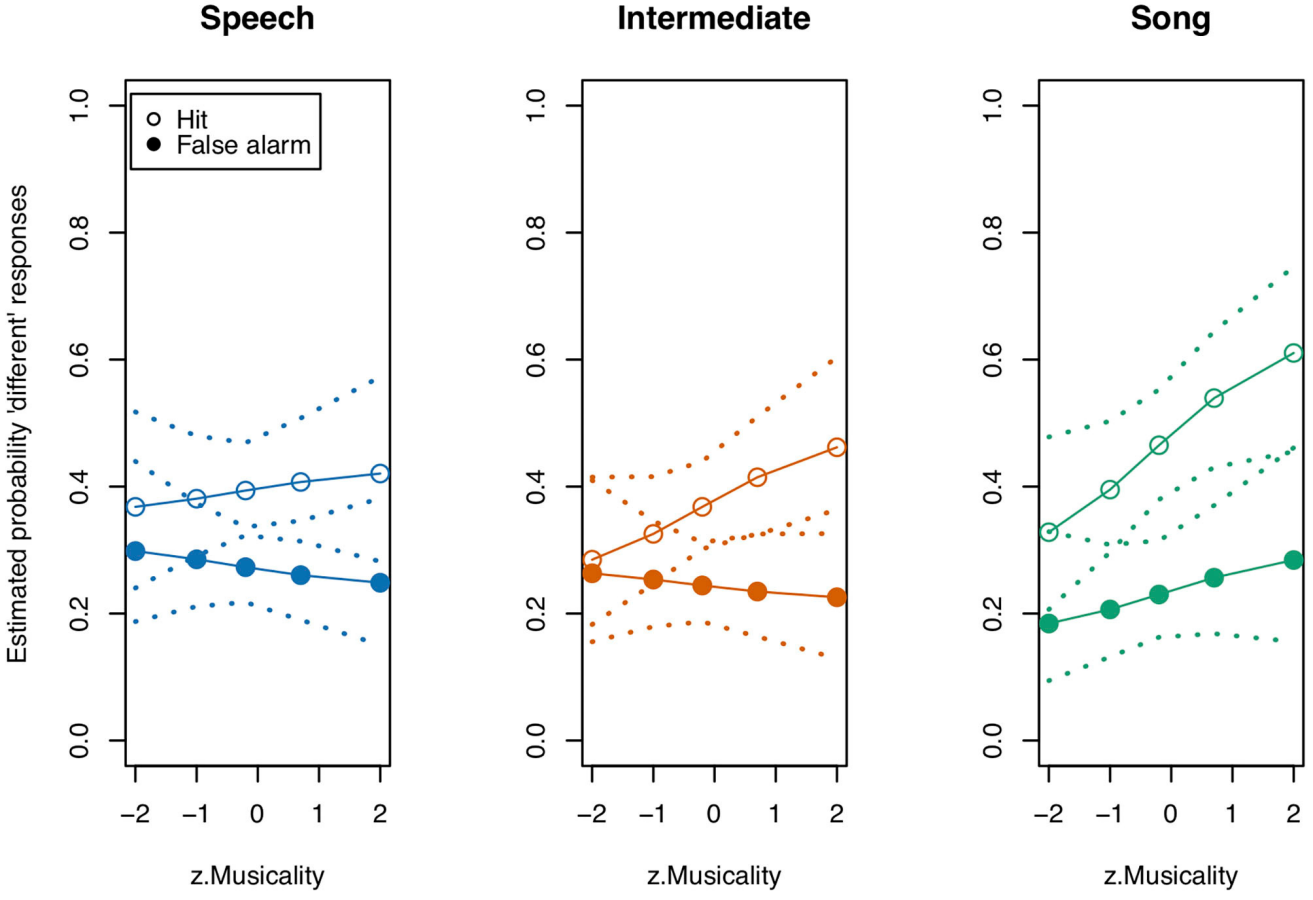
Model estimates for Musicality on the probability of detecting a deviant in study 2. Musicality had been assessed with the Gold-MSI and z-transformed for analysis. Empty circles: Hits, filled circles: False Alarms. Dotted lines represent 0.95 confidence intervals. Musicality showed the strongest impact for Intermediate stimuli. *N* = 38.

Although the full model (model coefficients see Table 5) revealed significance in comparison to the null model (χ^2^ = 40.959, df = 11, *p* < 0.001), likelihood ratio tests revealed no significance of the three-way interaction “Stimulus State:Variant:z.Musicality” (LRT = 0.485, AIC = 4886.652, df = 2, *p* = 0.785). There was also no significant effect of any of the two-way interactions “Stimulus State:Variant” (LRT = 3.795, AIC = 4886.447, df = 2, *p* = 0.450), “Stimulus State:z.Musicality” (LRT = 2.865, AIC = 4887.517, df = 1, *p* = 0.091), and “Variant:z.Musicality” (LRT = 4.142, AIC = 4886.794, df = 2, *p* = 0.126). Figures 4, 5 show the probability estimates based on the model. We could therefore not replicate our findings from study 1. However, based on the overall low values of d', we concluded that the task was too difficult for participants, which motivated study 3, in which we created a task of medium difficulty.

**Table 5:** Estimates of model coefficients (log odds), standard errors (SE), lower (CI_lower_) and upper (CI_upper_) 0.95 confidence intervals, z statistics (*z*-value) and associated *p*-values of the model fitted for study 2.

**Term**	**log odds**	**SE**	**CI_**lower**_**	**CI_**upper**_**	***z***	***p***
Intercept	−0.980	0.153	−1.292	−0.665	−6.411	
Stimulus state diff	0.550	0.169	0.231	0.887	3.247	0.001
Intermediate	−0.150	0.147	−0.437	0.142	−1.023	0.306
Song	−0.225	0.201	−0.614	0.160	−1.117	0.264
z.Musicality	−0.063	0.138	−0.333	0.207	−0.457	0.648
Stimulus state diff: Intermediate	0.044	0.228	−0.428	0.498	0.193	0.847
Stimulus state diff: Song	0.521	0.277	−0.022	1.072	1.885	0.059
Stimulus state diff:z.Musicality	0.117	0.145	−0.207	0.417	0.812	0.417
Intermediate:z.Musicality	0.012	0.125	−0.256	0.263	0.093	0.926
Song:z.Musicality	0.204	0.145	−0.066	0.478	1.410	0.159
Stimulus state diff: Intermediate:z.Musicality	0.125	0.186	−0.246	0.515	0.675	0.500
Stimulus state diff:Song:z.Musicality	0.032	0.191	−0.374	0.419	0.170	0.865

*The model estimated detection of a difference between original sound and target sound (reference level: Stimulus State same) as a function of Stimulus Variant (Speech, Song, Intermediate. Reference level: Speech) and Musicality as assessed by the GoldMSI questionnaire and z-transformed (that way the estimates refer to an individual with average musicality)*.

For the Anova, a qqplot and histogram of the residuals showed no obvious deviation from normality. The assumption of sphericity had not been violated (Mauchly's Test of Sphericity: *W* = 0.876, *p* = 0.092).

D-primes differed significantly between Variants [*F*_(2, 74)_ = 3.89, *p* = 0.02, generalised η^2^ = 0.05). Tukey-adjusted pairwise contrasts showed that the effect was driven by the contrast between Speech and Song (*t*_(74)_ = −2.638, *p* = 0.027). The contrast Intermediate–Song showed a trend [*t*_(74)_ = −2.105, *p* = 0.096], while the contrast Speech–Intermediate remained non-significant: *t*_(74)_ = −0.532, *p* = 0.855. For an overview of the distribution of d-primes as function of Stimulus Variant please see Supplementary Figure 3, for the raw hit and false alarm rates please see Supplementary Table 5.

## Study 3

Since the task changes we did in study 2 to replicate study 1 appeared to have lead to difficulties for our participants to be successful at all, we attempted to create a task of medium difficulty. To this end we allowed a slightly greater magnitude of deviation within target stimuli and changed the number of syllables per stimulus back to seven. Since the distractor sound in study 2–narrowband white noise bursts with different centre frequencies–might have interferred more strongly with discrete pitches, we created new distractor sounds based on female voices.

### Materials and Methods

#### Stimuli

The same stimulus contours as in study 1 were used, i.e., seven syllables per stimulus contour with a duration between 2 and 3 s. However, contours of which deviant syllables differed by more than six semitones from the original contour were removed (measured as the difference at 0.04 s from onset of a deviant syllable and the respective syllable of the original contour, using Praat). Thus, the mean absolute deviation was 1.6 semitones (SD = 1.9). We controlled the direction of deviation more precisely such that in study 3 a pitch interval from preceding syllable to target syllable always reversed the direction in comparison to the original stimulus, with no exception. This resulted in a pool of 45 contours in three variants (Song, Speech, Intermediate) each. As intermediate stimulus contour, step 5 of the song-speech continuum was used as in study 2. There were no significant differences between the three variants with regard to both directional (Median_Speech_: 1.06; Median_Intermediate_: 1.38; Median_Song_: 1.73; Kruskal Wallis Test: H = 0.35, df = 2, *p* = 0.84) and absolute semitone deviations (Median_Speech_: 4.10; Median_Intermediate_: 3.95; Median_Song_: 3.95; Kruskal Wallis Test: H = 0.68, df = 2, *p* = 0.71). Since the distractor sounds in study two consisted of narrowband noise chunks with stable frequency range for 0.2 to 0.3 s each they might have been perceived as being of stable pitch (however unprecise) by our participants. This way, study 2 would have been more like an n-back task with stronger interference for song variants. We therefore decided to create new distractor sounds consisting of a mixture of female voices. To this end, voices of six females were recorded singing one syllable [la:l] on tones one octave above the tones used for the actual (male voiced) stimuli. A Zoom H4n recording device (16 bit, 44.1 kHz) was used for these recordings. The procedure was the same as for creating the original stimuli. PitchTiers from all variants of the continuum (from speech-like to song-like) that were originally created as basis of the stimulus sounds (male voiced) were shifted up by one octave. Female recordings closest to the pitch of these octave-shifted PitchTiers were then adjusted in duration to the respective PitchTiers. Afterwards, PitchTier and respective recording were combined to keep the spectral information of the female recording along with the desired pitch contour. These syllables were then ramped (trapezoid). That way, a pool of syllables of six female voices was created. From this pool syllables were drawn randomly for each female voice to result in a stream of syllables of maximum 2 s duration. Streams of all six female voices were then mixed together to mono. This way, the distractors contained voices with intermixed syllables of all variants on the continuum. One unique distractor sound was assigned to each variant of each stimulus contour.

#### Procedure

Twenty one participants (16 females, mean age = 24.8 years, range 19 to 46 years) took part in the study. The same experimenter as in study 1 and 2 ran all participants.

The procedure was equal to that of study 2 except that the test of holistic vs. spectral hearing was left away. No participant needed more than 60 min to complete the study. None of the participants had to be excluded for statistical analysis.

#### Statistical Methods

We fitted a logistic GLMM with logit link function with the same parameters as in study 1. Interactions of random intercepts and random slopes were not included. Dummy coding was done using a custom-built function. Optimizer “bobyqa” with maximum 100.000 function iterations was used to assess the maximum likelihood.

The model showed singular fit due to boundary estimates in the random effects structure. Since the removal of random effects might lead to inflated Type I error rates and singular fit does not influence the estimates of the fixed effects, we decided to keep the random effects in the model.

Trials of blocks for which participants indicated a concentration level below 3 (on a 7-point Likert scale with 1 being lowest concentration) were excluded from the data. This was the case for 80 out of 2,520 trials, stemming from two blocks of one participant. Thus, 2,440 observations from 21 individuals remained as sample size for the model.

Model stability was assessed using a custom-built function. On this basis we did not obtain any influential cases, thus the model was stable (for ranges of parameter estimates of fixed effects please see Supplementary Table 6 and Supplementary Figure 6).

Collinearity was assessed as for study 1. There were no collinearity issues found.

Testing overdispersion revealed that the model was slightly underdispersed (χ^2^ = 2108.610, df = 2,419, *p* = 1, dispersion parameter = 0.87).

Full-null model-comparison and tests of fixed predictors were done using likelihood ratio tests as in study 1.

We derived 0.95-confidence intervals using the function “bootMer” (package “lme4”) with 1,000 parametric bootstraps.

After these analyses, we additionally did a repeated-measures Anova on classically calculated d-primes for informative purposes with the same parameters as in study 1 (Corrections for hit rates and false alarm rates of 0 and 1 had to be done for 5 of 63 cells for this analysis). *Post-hoc* tests were calculated using the package “lsmeans,” version 2.30-0 (Lenth, 2016).

### Results

#### Post-experiment Questionnaire

Three participants reported that they mentally visualised the contours in order to memorise them. Two participants reported using movements of their hands or feet for the same purpose. Six participants reported that they attempted to practice the stimulus silently during the distractor sound in order to not forget it. One participant guessed the study was about a comparison of prosody and melody.

#### Statistical Results

**Figure 6:**
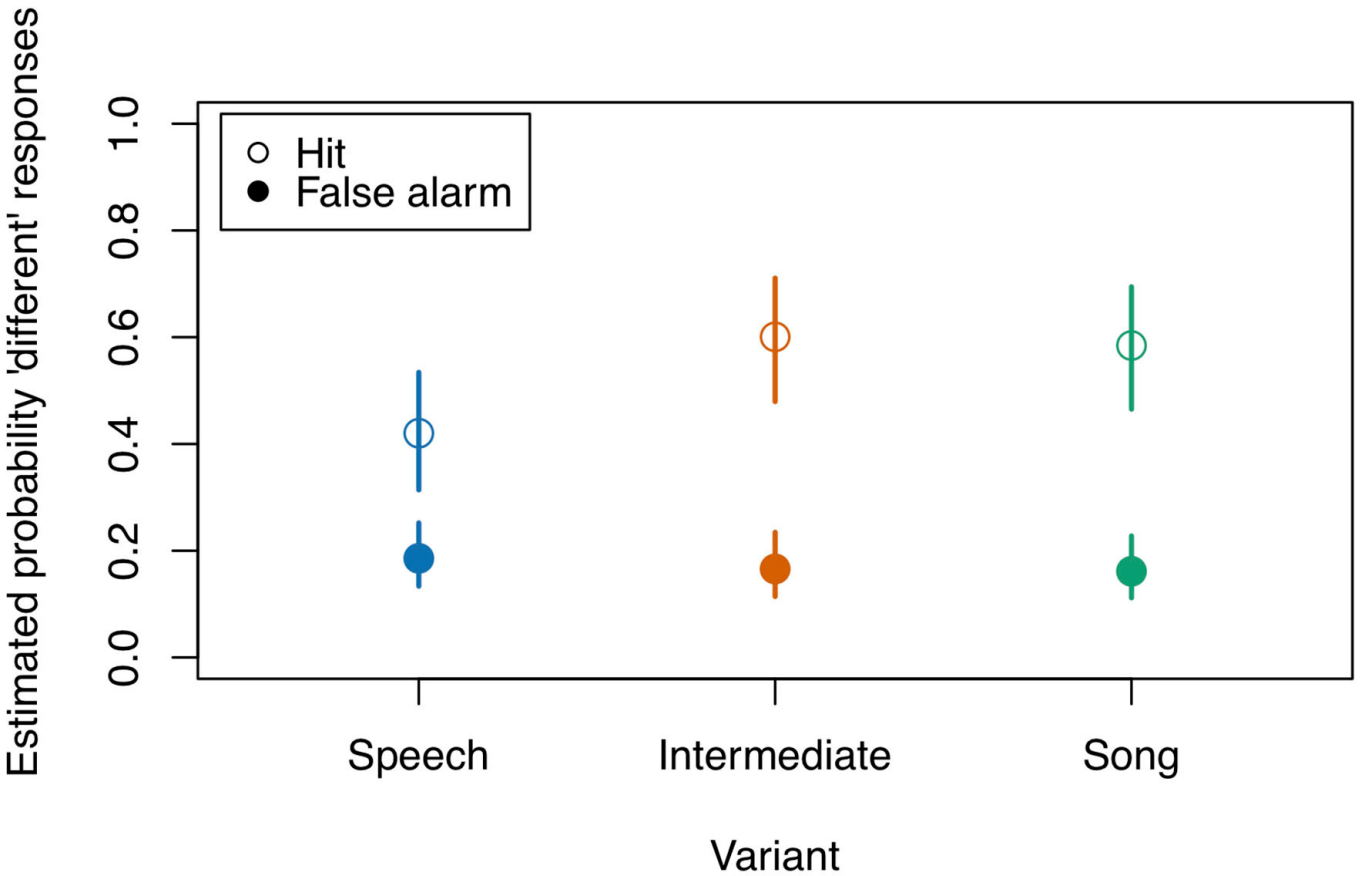
Model estimates for the probability of detecting a deviant in study 3 (see also Table 7). Empty circles: Hits, filled circles: False Alarms. Bars indicate 0.95 confidence intervals. *N* = 21.

After fitting the full model (model coefficients see Table 6) we compared it to the null model (which was lacking the predictors of interest). All predictors of interest together explained the data significantly better than the null model (χ^2^ = 67.342, df = 11, *p* < 0.001). Figures 6 and Table 7 show the probability estimates derived from our full model.

**Table 6:** Estimates of model coefficients (log odds), standard errors (SE), lower (CI_lower_) and upper (CI_upper_) 0.95 confidence intervals, z statistics (*z*-value) and associated *p*-values of the model fitted for study 3.

**Term**	**log odds**	**SE**	**CI_**lower**_**	**CI_**upper**_**	***z***	***p***
Intercept	−1.478	0.199	−1.921	−1.119	−7.413	
Stimulus state diff	1.156	0.224	0.725	1.642	5.167	0.000
Intermediate	−0.137	0.204	−0.576	0.258	−0.670	0.503
Song	−0.169	0.201	−0.581	0.236	−0.842	0.400
z.Musicality	−0.218	0.194	−0.596	0.118	−1.126	0.260
Stimulus state diff:Intermediate	0.867	0.247	0.370	1.390	3.519	0.000
Stimulus state diff: Song	0.832	0.249	0.339	1.320	3.342	0.001
Stimulus state diff:z.Musicality	0.456	0.221	0.024	0.897	2.063	0.039
Intermediate:z.Musicality	−0.030	0.188	−0.365	0.346	−0.157	0.875
Song:z.Musicality	−0.175	0.191	−0.531	0.169	−0.915	0.360
Stimulus state diff: Intermediate:z.Musicality	0.054	0.247	−0.407	0.534	0.217	0.828
Stimulus state diff: Song:z.Musicality	0.297	0.248	−0.160	−0.160	1.195	0.232

*The model estimated detection of a difference between original sound and target sound (reference level: Stimulus State same) as a function of Stimulus Variant (Speech, Song, Intermediate. Reference level: Speech) and Musicality as assessed by the GoldMSI questionnaire and z-transformed (that way the estimates refer to an individual with average musicality)*.

**Table 7:** Probability estimates and 0.95 confidence intervals for Hits and False Alarms as depicted in Figure 6.

	**Speech**			**Intermediate**			**Song**		
**Stimulus state**	**Estimate**	**CI_**lower**_**	**CI_**upper**_**	**Estimate**	**CI_**lower**_**	**CI_**upper**_**	**Estimate**	**CI_**lower**_**	**CI_**upper**_**
diff	0.420	0.314	0.534	0.601	0.479	0.711	0.584	0.495	0.694
same	0.186	0.134	0.252	0.166	0.114	0.234	0.161	0.112	0.228

*Derived from the R-package “effects,” version 4.1-0 (Fox et al., 2019)*.

**Figure 7:**
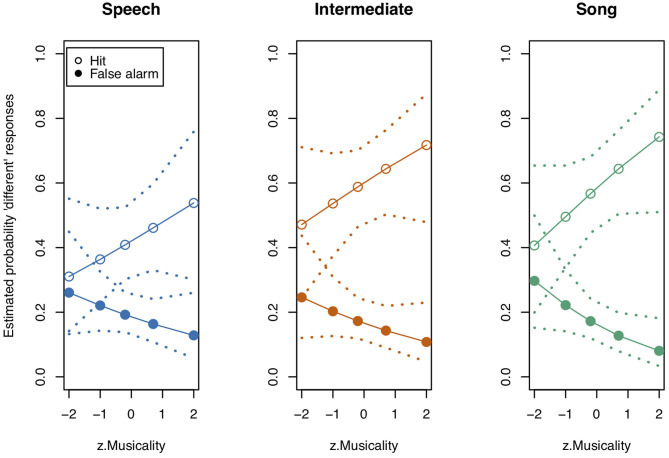
Model estimates for Musicality on the probability of detecting a deviant in study 3. Musicality had been assessed with the Gold-MSI and z-transformed for analysis. Empty circles: Hits, filled circles: False Alarms. Dotted lines represent 0.95 confidence intervals. *N* = 21.

Thereafter, we tested the effects of the interactions. In contrast to study 1, the three-way interaction “Stimulus State:Variant:z.Musicality” remained non-significant (LRT = 1.764, AIC = 2723.899, df = 2, *p* = 0.414). However, consistent with our hypothesis we found a significant interaction of “Stimulus State:Variant,” with Song and Intermediate enhancing memory with respect to Speech. Participants with a higher score in “Musicality” (assessed by the Gold-MSI) were better able to remember stimulus contours across all three Stimulus States (Song, Speech, Intermediate), suggested by a significant interaction of “z.Musicality” with “Stimulus State” (see Figure 7 and Table 8).

**Table 8:** Degrees of freedom (Df), Akaike Information Criteria (AIC), Likelihood Ratio test statistic (LRT), associated *p*-value [Pr(Chi)] and Nagelkerke's *R*^2^, based on likelihood ratio tests for two-way interactions of the model fitted in study 3.

**Term**	**Df**	**AIC**	**LRT**	**Pr(Chi)**	**Nagelkerke's *R*^**2**^**
Stimulus state:Variant	2	2729.686	15.174	<0.001	0.039
Stimulus State:z.Musicality	1	2725.351	8.839	0.003	0.035
Variant:z.Musicality	2	2714.512	<0.001	1.000	0.022

*Derived from the R-package “effects,” version 4.1-0 (Fox et al., 2019)*.

For the additional Anova, normality of residuals was assessed using a qqplot and histogram of the residuals. There was no obvious deviation from normality. The assumption of sphericity was not violated (Mauchly's Test of Sphericity: *W* = 0.896, *p* = 0.353). We found a significant difference between Variants [*F*_(2, 40)_ = 5.648, *p* = 0.007, η^2^ = 0.10).

Contrasts using Tukey-adjusted *p*-values showed that the differences that drove the effect of “Variants” were between Speech and Song [*t*_(40)_ = −2.476, *p* = 0.046], and between Speech and Intermediate [*t*_(40)_ = −3.206, *p* = 0.007], but not between Song and Intermediate [*t*_(40)_ = 0.731, *p* = 0.747]. For an overview of the distribution of d-primes as function of Stimulus Variant please see Supplementary Figure 4, for the raw hit and false alarm rates please see Supplementary Table 7.

## *Post-hoc* Rating Study

To clarify whether our results are better explained by stimulus-driven bottom-up effects or by top-down decision making based on the categories “song” and “speech,” we carried out an online rating study with a new sample of participants.

### Materials and Methods

#### Stimuli

We used all stimulus contours used in the three studies, with all three versions (Song, Speech, Intermediate), but without the distractor sounds. Additionally, we included the respective original Mandarin Chinese phrases that were the basis for the creation of our stimuli. These Mandarin phrases were lowpass-filtered with a cutoff of 600 Hz (100 Hz smoothing) to keep the pitch contours but reduce phonemic information. Stimuli had to be converted to mp3 format for technical reasons.

#### Procedure

The online study platform “Labvanced” (https://www.labvanced.com) was used for setting up the experiment and recruiting and testing participants. This platform operates under the General Data Protection Regulation of the European Union. Fifty seven participants took part in the study (38 females, mean age = 41.1 years, range 19 to 69 years). One participant had to be excluded because of not correctly filling out the consent form. All others gave informed consent. Participants were recruited world-wide. None of the participants stated that they spoke Mandarin Chinese.

First, a written instruction about the rating task was shown on the screen. Afterwards, participants had to do a headphone screening test implemented from the Labvanced study library to ensure that participants were wearing headphones. This test consisted of a three-alternative forced choice task where three sine tones of different loudness were presented per trial, with 12 trials in total. Only participants who successfully identified the quietest tones were allowed to continue. Subsequently, participants had to give informed consent that they understood the task, that their data would be used for analysis and that they were willing to participate. Afterwards, participants started the rating task, whereby each participant was presented with 13 randomly chosen stimuli from each of the four categories (Song, Speech, Intermediate, Lowpass-filtered Mandarin). The first 12 stimuli (three from each category) were considered training and not used in the analysis. During the display of a white screen, the stimulus was played. After playing of the stimulus was finished, a text (“How does it sound like?”) and two rating bars appeared. The rating bars corresponded to a 9-point Likert scale, but no numbers were displayed. The upper rating bar displayed “Song” (corresponding to a Likert rating of 1) and “Speech” (9) as extremes (Song-Speech rating), the lower rating bar showed “natural” (1) and “artificial” (9) as the extremes (Natural-Artificial rating). For each trial the rating button was initially set in the middle. After a two second delay, a button appeared to enable the participants to continue with the next trial. After the rating task, the Gold-MSI as well as additional questions on age, gender and languages spoken were presented. This was followed by a written debriefing stating the purpose of the study.

#### Statistical Analysis

We analysed both ratings (Song-Speech and Natural-Artificial) with Friedman tests, using the mean rating per participant and stimulus class. As *post-hoc* tests, we applied Wilcoxon signed-rank tests. For the Song-Speech rating we compared the following stimulus variants: lowpass Mandarin vs. Speech, Song vs. Speech, Speech vs. Intermediate, and Song vs. Intermediate. We also calculated the differences in ratings for Speech vs. Intermediate and Song vs. Intermediate and compared those using a Wilcoxon test. For the Artificial-Natural rating, we compared the following stimulus variants: lowpass Mandarin vs. Speech, and Song vs. Speech.

### Results

The results showed that when asked explicitly, participants rated all three stimulus classes used in our studies (Song, Speech, Intermediate) with a tendency towards artificially sounding, while the lowpass-filtered Mandarin was rated with a tendency towards natural sounding (see Supplementary Figure 7). Because of the significant Friedman test for the Natural-Artificial rating (χ^2^ = 60.367, df = 3, *p* < 0.001), we conducted Wilcoxon tests for pairwise comparison. Lowpass Mandarin was rated significantly more towards natural sounding than Speech (Median_lowpass Mandarin_ = 3.725, Median_Speech_ = 7.05, *z* = 6.046, *N* = 53, *p* < 0.001) while both Song and Speech did not differ significantly in their ratings towards artificially sounding (Median_Song_ = 7.3, Median_Speech_ = 7.05, *z* = 0.188, *N* = 41, *p* = 0.855; the Median for Intermediate was 7.0). These results suggest that although the stimuli sounded artificial, there was no great difference in this respect between stimulus categories.

For the Song-Speech rating, all three stimulus classes used in our studies (Song, Speech, Intermediate) tended to be rated as more song-like, while the lowpass-filtered Mandarin was rated as more speech-like (see Supplementary Figure 8). Since the Friedman test was significant (χ^2^ = 125.330, df = 3, *p* < 0.001), we could conduct *post-hoc* Wilcoxon tests. Among the three stimuli used in our studies, Song was rated significantly more song-like than Intermediate (Median_Song_ = 2.15, Median_Intermediate_ = 2.5, *z* = 2.692, *N* = 48, *p* = 0.006) and Intermediate was rated significantly more song-like than Speech (Speech vs. Intermediate: Median_Speech_ = 3.3, Median_Intermediate_ = 2.5, *z* = 5.476, *N* = 49, *p* < 0.001). Song and Speech ratings differed significantly (Median_Song_ = 2.15, Median_Speech_ = 3.3, *z* = 5.436, *N* = 48, *p* < 0.001) as did the ratings for lowpass Mandarin and Speech (Median_lowpass Mandarin_ = 8.6, Median_Speech_ = 3.3, *z* = 6.451, *N* = 55, *p* < 0.001). The difference between ratings for Intermediate and Speech were significantly larger than those for ratings between Intermediate and Song (Median_Speech−Intermediate_ = 0.75, Median_Intermediate−Song_ = 0.1, *z* = 4.952, *N* = 50, *p* < 0.001). These results suggest that the memory advantage we observed in studies 1 and 3 was likely stimulus-driven rather than mediated by a top-down decision making guided by a “song vs. speech” decision.

## Discussion

In the current studies we tested the hypothesis that the discrete pitches typifying song are a spectrotemporal property optimised for auditory memory. We thus predicted that vocal phrases are remembered better when consisting of discrete (song-like) pitches than when consisting of gliding (speech-like) pitches. To this end we conducted three studies in a same-different paradigm, using a forced-choice task where song-like, speech-like and intermediate stimuli were presented. To vary task difficulty among studies, we modified stimulus length, used a different type of distractor sound in each study, and slightly modified the difficulty of discrimination in each task. The results of study 1 are consistent with our hypothesis, showing that the use of discrete pitches in an auditory stimulus modelled after a vocal phrase indeed enhances memory of that stimulus. In study 2 we were not able to replicate these results, possibly due to overall task difficulty. In study 3 we replicated the effect of memory advantage for stimuli of discrete pitches that we found in study 1. Intermediate stimuli (partially discretised) could be remembered as well as song-like stimuli in studies 1 and 3. We also found that participants with more musical experience remembered the stimuli better, in both study 1 and study 3. In study 1 this effect occurred as a stronger advantage for more musically experienced participants for song-like stimuli, compared to the other stimuli. In study 3 we found that participants with more musical experience remembered all three types of stimuli (Song, Speech, Intermediate) better than participants with less musical experience. Our results are thus consistent with the hypothesis that discrete pitches are a spectrotemporal property aiding auditory memory (“song memory advantage” hypothesis), and do not support the alternative “experience advantage” hypothesis: that the abundance of speech perception and production throughout the human lifespan would make gliding pitch more salient for auditory memory than discrete pitch.

Our results indicate that one fundamental design feature of music, discrete pitches, has cognitive effects that go beyond surface-level perception. Our results cannot be based on timbre differences, since all stimuli were based on recorded syllables from the same male voice. Neither can the effects be explained by differences in rhythmic structure, isochrony or meter since all three versions of our stimuli (Song, Speech and Intermediate) used the same syllable timing. Syllable durations were based on natural speech prosody and were additionally occasionally lengthened to have at least 250 ms duration, making them non-isochronous. Finally, we used the same set of pitch contours, that is the global upwards and downwards movement of the pitch, for all three stimulus types. Therefore, we conclude that one of the design features of song (and music more generally)–the use of discrete pitches–has a significant cognitive effect on auditory memory, and is not a non-functional side-effect of speech prosody.

We used Mandarin Chinese as the pitch contour template for our stimuli to minimise the influence of familiarity to our participants, who were not experienced with tonal languages. Although non-tonal language speakers are worse than native speakers of Mandarin in processing rapid pitch changes of lexical tone (Krishnan et al., 2010) and musical expertise may facilitate lexical tone processing (Alexander et al., 2005; Chandrasekaran et al., 2009), we emphasise that our stimulus creation process favoured the maintenance of sentence-level prosody in the stimuli over lexical tones. Moreover, the overall pitch contour was the same across all three stimulus types (see Supplementary Figures 9–14 for three sample stimuli and Supplementary Figures 15, 16 for a comparison to natural song and speech pitch contours). Sentence-level prosody of Mandarin Chinese has been shown to be unfamiliar but still behaviourally and neurally processable by non-tonal language speakers (Tong et al., 2005). It is therefore unlikely that our effects are related to difficulty in following rapid pitch changes in the speech prosody stimuli and are rather explained by a memory advantage for discrete pitch.

Our *post-hoc* rating study showed that when explicitly asked to rate the stimuli used in studies 1 to 3 (Song, Speech, Intermediate) as well as lowpass-filtered Mandarin phrases as more song-like or more speech-like, the former were rated as more song-like while the lowpass-filtered Mandarin phrases were rated as more speech-like. Lowpass-filtered Mandarin was also rated as more natural sounding, while Song, Speech and Intermediate stimuli were rated as more artificially sounding, with no significant differences among the latter. Even though the nature of the rating study was different from studies 1 to 3 (which did not include lowpass-filtered Mandarin phrases nor explicit mentioning of “song” and “speech” categories), this results suggests that our stimuli, although based on recordings of natural vocalisations, might not have represented natural speech and song very well. The memory effects we observed are therefore probably based on bottom-up processing of the pitch differences rather than categorical top-down processing. It is possible that the fact that including the lowpass-filtered Mandarin phrases induced that the Song, Speech and Intermediate stimuli were perceived as very similar and thus biased towards a song-like rating. However, among the three stimulus variants used in studies 1 to 3, Song stimuli were still rated as most song-like, and Speech stimuli as least song-like, with Intermediate stimuli in between, all differing significantly. It thus seems possible that actual natural speech and song might induce a stronger difference in auditory memory, or that the effect could be replicated with non-vocal stimuli as well. These questions could be addressed by future studies.

We were unable replicate our findings in study 2. D-primes were on average lowest for this study in comparison to studies 1 and 3. It seems therefore likely that the task in study 2 was too difficult for our participants, which masked any differences between stimulus variants. This could be explained by changes in the distractor stimuli we introduced to increase the difficulty of study 2. In comparison to the other two studies, we used narrowband white noise with different, variable centre frequencies as distractor sounds, while study 1 used simple pink noise bursts, and study 3 used a “cocktail party” mixture of female voices. We also decreased the pitch deviation of the target from the template sound among the studies (study 1: mean = 3.8 semitones, SD = 5.0; study 2: mean = 1.1 semitones, SD = 1.4; study 3: mean = 1.6 semitones, SD = 1.9). Moreover, we increased the number of syllables in each stimulus to 10 in study 2 (rather than 7 as in studies 1 and 3). This increased the duration of each stimulus contour to 3 to 4 s (studies 1 and 3: duration between 2 and 3 s). These three differences conspired to make study 2 a very challenging task for our participants.

Narrowband white noise stimuli with different centre frequencies can lead to a perception of discrete tones (see Hesse, 1982). This “pitchlike” quality of the distractor sounds in study 2 may have interfered most with discrete-pitched song stimuli and least with speech prosody stimuli. However, d-primes were also low for speech prosody stimuli in this study. Moreover, in study 3 we included discrete-pitched and gliding female voices as distractor sounds, which should have been most distracting throughout the three studies due to the close similarity to our target stimuli. In study 3 we nonetheless replicated the findings of study 1. The distractor sounds used in study 2 might therefore explain the low performance of our participants to only a small degree. Pitch deviations in target sounds however were smallest for study 3 in comparison to studies 1 and 2. Winkler et al. (2002) showed that auditory sensory memory representations are less stable for smaller pitch deviations after a 10-s silent interval. In line with these findings, visual inspection of the data of study 3, the study where our participants performed sufficiently well, and where pitch deviations were tightly controlled, shows that greater pitch deviation slightly enhanced d-primes (see Supplementary Figure 17). Nonetheless, it also shows that performance remained quite high for small pitch deviations. Small pitch deviations therefore seem insufficient to explain the low performance of our participants in study 2. Finally, the number of syllables per stimulus was 10 in study 2 (vs. 7 in the other studies). Williamson et al. (2010) showed in a tone sequence memory test that performance approached chance level when sequences consisted of more than seven tones. It seems plausible that this was the case for our study 2 participants as well. If this is indeed the main explanation for the low performance of our participants in study 2, it might suggest that our stimuli were remembered element-wise rather than as a global pitch contour. This seems natural concerning the task was to detect pitch deviations in a single syllable. Future research should investigate limits of auditory memory capacity for syllables with discrete and gliding pitch more thoroughly and systematically.

We found that participants with higher musical experience remembered the stimuli better, in both study 1 and study 3. Among the three stimulus types this advantage was strongest for song stimuli in study 1. In study 3 there was no significant difference for the effect of musical experience between the three stimulus types. This suggests that the advantage musically experienced people show occurs for song as well as for speech prosody stimuli and the intermediate stimulus class, not specifically for song stimuli only. This is in line with cross-over effects of musical training into the linguistic domain (Patel and Morgan, 2017). Musicians have also be shown to process rapid pitch changes better than non-musicians (Chandrasekaran et al., 2009). Although the pitch changes in our stimuli were slower than in Mandarin Chinese lexical tone, this might have influenced the detection of deviants in musicians. A meta-analysis by Talamini et al. (2017) also suggested that musicians show a general advantage over non-musicians in auditory working memory, in both tonal stimuli and verbal stimuli, with tonal stimuli showing the largest effect size. However, our data show that participants with lower self-assessed musicality also remember vocal phrases better when they consist of discrete pitches. This indicates that discrete pitches are processed similarly in auditory memory by both musicians and non-musicians, and that no musical training is necessary to obtain an advantage for discretised pitch. Our stimuli were not based on western musical scales (we used a Bohlen-Pierce scale as our model). The memory advantage for discretised pitch might be stronger, and might be greater in musicians, if major and minor western scales are used. Based on these results, we conclude that musical training is not a necessary prerequisite for a memory advantage for discrete pitches, and that the average day-to-day exposure to music of any hearing person in modern society may be enough to elicit this memory advantage.

An unexpected finding was that vocal phrases intermediate between song- and speech-like intonation, that is with syllables with partially stable and partially gliding pitch, were remembered equally well as song-like (fully discrete) phrases. We derived the category boundary between speech prosody and song for study 1 (based on a 9-step continuum, using a staircase procedure with two-alternative forced choice categorisation as speech/song, see Kingdom and Prins, 2016) by estimating the point of subjective equality for one subject (author FH). For study 1 we then used the stimulus closest to that boundary (step 6), and later we used one step closer to Speech (step 5) as the intermediate stimulus in studies 2 and 3. We expected that stimuli falling between clear song and speech categories would elicit a moderate memory advantage at best, but in fact performance was comparable to the clear song category. However, the *post-hoc* rating study showed that the ratings differed significantly between all three stimulus variants used in studies 1 to 3, with Song being rated more song-like than Intermediate, and Intermediate being rated more song-like than Speech. This suggests that the intermediate stimulus was indeed perceived as being between Song and Speech stimuli. Although the differences between ratings for Speech and Intermediate were significantly greater than those between Song and Intermediate, it remains debatable how well this explains the comparable memory effects for Song and Intermediate stimuli. To further probe the finding that our intermediate stimuli showed similar memory effects to our song stimuli, although being significantly discriminated from both speech prosody stimuli and song stimuli, it would be useful in future to use adaptive psychoacoustic methods to choose the stimulus material. In doing so, the stimulus space spanning between speech prosody and song could be explored in an efficient way. This could be informative about the extent that speech and song form two perceptual categories, based on pitch contour differences, potentially revealing ambiguous stimuli individually for each participant. Contextual cues that would change the categorical interpretation of these ambiguous stimuli as song- or speech-like would allow experimenters to isolate the influence of bottom-up perceptual vs. top-down categorical processes on auditory memory.

Overall, our findings indicate that song-like pitch trajectories, consisting of discrete pitches, yield a memory advantage over speech-like stimuli with gliding pitch. Since discrete pitch is a characteristic feature of song in comparison to speech across cultures, this suggests that the spectrotemporal structure of song as a vocal signal yields a behavioural outcome–remembering vocal sequences–that differs quantitatively from that elicited by speech intonation. The auditory memory system seems to make different use of these two signal types, or alternatively, there might be two distinct subsystems for memory based on pitch, echoing the distinction that Schulze et al. (2018) proposed for the subvocal rehearsal of verbal material (phonological loop) and tonal material (tonal loop), but on the level of pitch memory itself. In line with this, Zatorre and Baum (2012) suggested a distinction between coarse-grained and fine-grained mechanisms for auditory pitch contour analysis, in which fine-grained mechanisms are used exclusively to perceive musical sounds. Consistent with this hypothesis, cortical representations of pitch have been found to differ between speech and song (Merrill et al., 2012; Tierney et al., 2013). Across our stimulus types the memory advantage for song and intermediate stimuli, relative to speech prosody stimuli, is probably related to fine-grained pitch analysis, because the overall up-and-down movement of the pitch contour was kept constant. This is because each song pitch contour was matched with an equivalent intermediate- and speech-stimulus pitch contour, with only the pitch trajectory within each syllable differing (gliding, intermediate, or discrete, respectively).

If vocal phrases were remembered better when produced with discretised pitches, why do humans not sing all the time? Focusing just on pitch in speech and song, one could argue that speech prosody functions primarily in a context of temporal proximity, for example in a turn-taking context that is more typical of speech than music (Cross et al., 2013). This might make exact reproduction of pitch unnecessary. The use of phonetically distinct words to convey information about concepts, objects etc. and the ability to remember this information, independent of pitch, might further relax the necessity to remember (gliding) speech prosodic contours exactly. Even in tonal languages, the phonetic information conveyed by pitch rests mainly on pitch contour (e.g., falling, rising) and very rough pitch range (e.g., high, low) differences rather than on exact pitch reproduction.

However, vocal phrases that need to be remembered for longer durations and more precisely might need to rely on another structural layer beyond such coarse-grained intonation contour, namely specific relations among pitches. In fact, there is a long tradition of using sung passages to learn, remember, and pass down verbal information, from the sung works of Homer (initially orally transmitted), to Viking sagas, folk ballads, or religious texts such as the Rig Veda. This use of sung text is both ancient historically, and widespread among human cultures. Indeed indigenous Australians use melodic contours to map landscapes and pass this information orally, and such “songlines” cross language barriers and are at the same time deeply connected to myth and group identity (see e.g., Norris and Harney, 2014). This suggests that discrete pitches have been used to remember vocal phrases across time and cultures. Thus, the emergence of discrete pitches may result from a necessity to remember vocal phrases, making use of absolute and relative pitch perception. If this was the case, it would suggest that discrete pitches emerged as a fundamental feature of music to solve a specific cognitive problem, rather than a by-product of speech prosody [as argued by Pinker (1997)].

In summary, we found that discrete pitches lead to a memory advantage for auditory sequences modelled after vocal phrases. This effect occured in both more and less musically experienced people, although more musically experienced people showed a better general memory for vocal phrases (independent of gliding or discrete pitch). Our results show that at least one spectrotemporal design feature distinguishing music from spoken language has clear cognitive and behavioural consequences, making it unlikely to be an epiphenomenon of speech prosody. Rather, discrete pitch seems to be a feature enhancing auditory memory. Our results open the door for future studies to further explore the memory advantage of song over speech. Comparative studies involving non-human animals, including additional design features of music, might shed a light on their evolutionary origin.

## Data Availability Statement

The original contributions presented in the study are included in the article/Supplementary Material, further inquiries can be directed to the corresponding author.

## Ethics Statement

The studies involving human participants were reviewed and approved by Ethics Committee of the University of Vienna. The patients/participants provided their written informed consent to participate in this study.

## Author Contributions

FH and WF contributed to conception and design of the study. FH recruited and tested the participants. Under guidance of CQ and WF, FH performed the statistical analysis and drafted the manuscript. All authors contributed to manuscript revision, read, and approved the submitted version.

## Conflict of Interest

The authors declare that the research was conducted in the absence of any commercial or financial relationships that could be construed as a potential conflict of interest.

## Footnotes

^1^Mandarin phrases were downloaded from http://www.101languages.net/chinese/chinese-vocabulary-lessons/ (provided by 50languages.com under the creative commons license CC BY-NC-ND 3.0 US, https://creativecommons.org/licenses/by-nc-nd/3.0/us/).

^2^The experiment was prepared in both German and English. All but one participant chose the German version.

## References

[B1] Alexander J. A. Wong P. C. M. Bradlow A. R. (2005). Lexical tone perception in musicians and non-musicians, in Ninth European Conference on Speech Communication and Technology (Lisbon).

[B2] Arnold K. M. Jusczyk P. W. (2002). Text-to-tune Alignment in Speech and Song. Speech and Prosody. p. 135–138. Available online at: https://www.isca-speech.org/archive/sp2002/papers/sp02_135.pdf (accessed November 26, 2020).

[B3] Bates D. Maechler M. Bolker B. Walker S. (2015). lme4: Linear Mixed-Effects Models Using Eigen and S4, Version 1.1-21. [Computer Software]. Retrieved from: https://CRAN.R-project.org/package=lme4

[B4] Boersma P. Weenink D. (2017). Praat: Doing Phonetics by Computer, Version 6.0.36. [Computer Software]. Retrieved from: http://www.praat.org/

[B5] Bradley E. (2013). Pitch perception in lexical tone and melody. Rev. Res. Hum. Learn. Music 1, 1–26. 10.6022/journal.rrhlm.2013002

[B6] Bregman A. S. (1994). Auditory Scene Analysis: the Perceptual Organization of Sound. Cambridge, MA: MIT Press 10.1121/1.408434

[B7] Bregman M. R. Patel A. D. Gentner T. Q. (2016). Songbirds use spectral shape, not pitch, for sound pattern recognition. Proc. Natl. Acad. Sci. U.S.A. 113, 1666–1671. 10.1073/pnas.1515380113 26811447 PMC4760803

[B8] Buchsbaum B. R. (2016). Working memory and language, in Neurobiology of Language, eds Hickok G. Small S. L. (Elsevier), 863–875. 10.1016/B978-0-12-407794-2.00069-9

[B9] Chandrasekaran B. Krishnan A. Gandour J. T. (2009). Cortical processing of pitch contours. Brain Lang. 108, 1–9. 10.1016/j.bandl.2008.02.001 18343493 PMC2670545

[B10] Corretge R. (2012). Praat Vocal Toolkit: A Praat Plugin With Automated Scripts for Voice Processing. [Computer Software]. Retrieved from: http://www.praatvocaltoolkit.com

[B11] Cross I. Fitch W. T. Aboitiz F. Iriki A. Jarvis E. D. Lewis J. (2013). Culture and evolution, in Language, Music, and the Brain, ed Arbib M. A. (Cambridge, MA: MIT Press), *541–562* 10.7551/mitpress/9780262018104.003.0021

[B12] Darwin C. (1871). The Descent of Man, and Selection in Relation to Sex. London: J. Murray 10.5962/bhl.title.2092

[B13] Dobson A. J. (2002). An Introduction to Generalized Linear Models. Boca Raton: Chapman and Hall/CRC 10.1201/9781420057683

[B14] Fitch W. T. (2006). The biology and evolution of music: a comparative perspective. Cognition 100, 173–215. 10.1016/j.cognition.2005.11.009 16412411

[B15] Fitch W. T. Jarvis E. D. (2013). Birdsong and other animal models for human speech, song, and vocal learning, in Language, Music, and the Brain, ed Arbib M. A. (Cambridge, MA: MIT Press), 499–540. 10.7551/mitpress/9780262018104.003.0020

[B16] Forstmeier W. Schielzeth H. (2011). Cryptic multiple hypotheses testing in linear models: overestimated effect sizes and the winner's curse. Behav. Ecol. Sociobiol. 65, 47–55. 10.1007/s00265-010-1038-5 21297852 PMC3015194

[B17] Fox J. Weisberg S. Price B. Friendly M. Hong J. Andersen R. (2019). Package “Effects,” Version 4.1-0. [Computer Software]. Retrieved from: https://CRAN.R-project.org/package=effects

[B18] Fox J. Weisberg S. Price B. Friendly M. Hong J. Andersen R. (2019). Package “Effects.”

[B19] Fritz J. Poeppel D. Trainor L. Schlaug G. Patel A. D. Peretz I. (2013). The neurobiology of language, speech, and music, in Music, Language, and the Brain, ed Arbib M. A. (Cambridge, MA: MIT Press), 417–459. 10.7551/mitpress/9780262018104.003.0017

[B20] Hesse H.-P. (1982). The judgment of musical intervals, in Music, Mind, and Brain, ed Manfred C. (Berlin: Springer), 217–225. 10.1007/978-1-4684-8917-0_11

[B21] Hickok G. Buchsbaum B. Humphries C. Muftuler T. (2003). Auditory-motor interaction revealed by fMRI: Speech, music, and working memory in area Spt. J. Cogn. Neurosci. 15, 673–682. 10.1162/089892903322307393 12965041

[B22] Kingdom F. A. A. Prins N. (2016). Psychophysics: A Practical Introduction, 2nd Edn. London: Academic Press.

[B23] Krishnan A. Gandour J. T. Smalt C. J. Gavin M. (2010). Language-dependent pitch encoding advantage in the brainstem is not limited to acceleration rates that occur in natural speech. Brain Lang. 114, 193–198. 10.1016/j.bandl.2010.05.004 20570340 PMC2913296

[B24] Krohn K. I. Brattico E. Välimäki V. Tervaniemi M. (2007). Neural representations of the hierarchical scale pitch structure. Music Percept. 24, 281–296. 10.1525/mp.2007.24.3.281

[B25] Lenth R. (2016). lsmeans: Least-Squares Means, Version 2.30-0. [Computer Software]. Retrieved from: https://CRAN.R-project.org/package=lsmeans

[B26] Levinson S. C. (2013). Cross-cultural universals and communication structures, in Language, Music, and the Brain, Vol. 10, ed Arbib M. A. (Cambridge, MA: MIT Press), 67–81. 10.7551/mitpress/9780262018104.003.0003

[B27] Lindblom B. Sundberg J. (2014). The human voice in speech and singing, in Springer Handbook of Acoustics, ed Rossing T. D. (New York, NY: Springer New York), 703–746. 10.1007/978-1-4939-0755-7_16

[B28] Lloyd E. A. Gould S. J. (2017). Exaptation revisited: changes imposed by evolutionary psychologists and behavioral biologists. Biol. Theory 12, 50–65. 10.1007/s13752-016-0258-y

[B29] Mampe B. Friederici A. D. Christophe A. Wermke K. (2009). Newborns' cry melody is shaped by their native language. Curr. Biol. 19, 1994–1997. 10.1016/j.cub.2009.09.064 19896378

[B30] Mehr S. A. Singh M. Knox D. Ketter D. M. Pickens-Jones D. Atwood S.. (2019). Universality and diversity in human song. Science 366:eaax0868. 10.1126/science.aax0868 31753969 PMC7001657

[B31] Merrill J. (2013). Song and speech perception–evidence from fMRI, lesion studies and musical disorder (PhD Thesis). Leipzig: Max Planck Institute for Human Cognitive and Brain Sciences.

[B32] Merrill J. Sammler D. Bangert M. Goldhahn D. Lohmann G. Turner R.. (2012). Perception of words and pitch patterns in song and speech. Front. Psychol. 3:76. 10.3389/fpsyg.2012.00076 22457659 PMC3307374

[B33] Müllensiefen D. Gingras B. Musil J. Stewart L. (2014). The musicality of non-musicians: an index for assessing musical sophistication in the general population. PLoS ONE 9:e101091. 10.1371/journal.pone.0089642 24586929 PMC3935919

[B34] Nieuwenhuis R. te Grotenhuis M. Pelzer B. (2012). Influence.ME: tools for detecting influential data in mixed effects models. R J. 4, 38–47. 10.32614/RJ-2012-011

[B35] Norris R. P. Harney B. Y. (2014). Songlines and navigation in wardaman and other australian aboriginal cultures. J. Astron. Hist. Herit. 17, 1–15. Available online at: https://www.researchgate.net/publication/261512575_Songlines_and_Navigation_in_Wardaman_and_other_Australian_Aboriginal_Cultures

[B36] Oh J. Fitch W. T. (2018). Experimenter_GUI, Version 0.1. [Computer Software]. Retrieved from: https://github.com/jinook0707/Experimenter

[B37] Patel A. D. Morgan E. (2017). Exploring cognitive relations between prediction in language and music. Cogn. Sci. 41, 303–320. 10.1111/cogs.12411 27665745

[B38] Paulmann S. (2016). The neurocognition of prosody, in Neurobiology of Language, eds Hickok G. Small S. L. (London: Elsevier), 1109−1120. 10.1016/B978-0-12-407794-2.00088-2

[B39] Pinker S. (1997). How the Mind Works (1st ed). New York: WW Norton.

[B40] Pisanski K. Cartei V. McGettigan C. Raine J. Reby D. (2016). Voice modulation: a window into the origins of human vocal control? Trends Cogn. Sci. 20, 304–318. 10.1016/j.tics.2016.01.002 26857619

[B41] Poeppel D. (2011). Genetics and language: a neurobiological perspective on the missing link (-ing hypotheses). J. Neurodev. Disord. 3, 381–387. 10.1007/s11689-011-9097-0 22052160 PMC3261270

[B42] R Core Team (2019). R: A Language and Environment for Statistical Computing. Vienna: R Foundation for Statistical Computing.

[B43] Schneider P. Sluming V. Roberts N. Scherg M. Goebel R. Specht H. J.. (2005). Structural and functional asymmetry of lateral Heschl's gyrus reflects pitch perception preference. Nat. Neurosci. 8, 1241–1247. 10.1038/nn1530 16116442

[B44] Schulze K. Koelsch S. Williamson V. (2018). Auditory working memory, in Springer Handbook of Systematic Musicology, ed Bader R. (Berlin: Springer), 461–472. 10.1007/978-3-662-55004-5_24

[B45] Seifert U. Verschure P. F. M. J. Arbib M. A. Cohen A. J. Fogassi L. Fritz T. H. (2013). Semantics of internal and external worlds, in Language, Music, and the Brain: A Mysterious Relationship, ed Arbib M. A. (Cambridge, MA: MIT Press), 203–232. 10.7551/mitpress/9780262018104.003.0008

[B46] Semal C. Demany L. Ueda K. Hallé P. (1996). Speech versus nonspeech in pitch memory. J. Acoust. Soc. Am. 100, 1132–1140. 10.1121/1.416298 8759966

[B47] Singmann H. Bolker B. Westfall J. Aust F. (2020). afex: Analysis of Factorial Experiments, Version 0.26-0. [Computer Software]. Retrieved from: https://CRAN.R-project.org/package=afex

[B48] Talamini F. Altoè G. Carretti B. Grassi M. (2017). Musicians have better memory than nonmusicians: a meta-analysis. PLoS ONE 12, 1–21. 10.1371/journal.pone.0186773 29049416 PMC5648224

[B49] ten Cate C. Rowe C. (2007). Biases in signal evolution: learning makes a difference. Trends Ecol. Evolut. 22, 380–387. 10.1016/j.tree.2007.03.006 17379354

[B50] Tierney A. Dick F. Deutsch D. Sereno M. (2013). Speech versus song: multiple pitch-sensitive areas revealed by a naturally occurring musical illusion. Cereb. Cortex 23, 249–254. 10.1093/cercor/bhs003 22314043 PMC3539450

[B51] Tillmann B. Albouy P. Caclin A. (2015). Congenital amusias, Handbook of Clinical Neurology, 1st ed., Vol. 129, eds Aminoff M. J. Boller F. Swaab D. F. (Amsterdam: Elsevier B.V), 589–605. 10.1016/B978-0-444-62630-1.00033-0 25726292

[B52] Tong Y. Gandour J. Talavage T. Wong D. Dzemidzic M. Xu Y.. (2005). Neural circuitry underlying sentence-level linguistic prosody. NeuroImage 28, 417–428. 10.1016/j.neuroimage.2005.06.002 16006150

[B53] Trehub S. E. (2013). Communication, music and language in infancy, in Language, Music, and the Brain: A Mysterious Relationship, ed Arbib M. A. (Cambridge, MA: MIT Press), 463–479. 10.7551/mitpress/9780262018104.003.0018

[B54] Ueda K. (2004). Short-term auditory memory interference: the Deutsch demonstration revisited. Acoust. Sci. Technol. 25, 457–467. 10.1250/ast.25.457

[B55] Verhoef T. Kirby S. Padden C. (2011). Cultural emergence of combinatorial structure in an artificial whistled language, in Proceedings of the 33rd Annual Meeting of the Cognitive Science Society (2008) (Washington, DC), 483–488. Available online at: https://escholarship.org/uc/item/9rh914kc (accessed November 26, 2020).

[B56] Williamson V. J. Baddeley A. D. Hitch G. J. (2010). Musicians' and nonmusicians' short-term memory for verbal and musical sequences: comparing phonological similarity and pitch proximity. Memory Cognit. 38, 163–175. 10.3758/MC.38.2.163 20173189

[B57] Winkler I. Korzyukov O. Gumenyuk V. Cowan N. Linkenkaer-Hansen K. Ilmoniemi R. J.. (2002). Temporary and longer term retention of acoustic information. Psychophysiology 39, 530–534. 10.1017/S0048577201393186 12212645

[B58] Zahorik P. Brungart D. S. Bronkhorst A. W. (2005). Auditory distance perception in humans : a summary of past and present research. Acta Acustica United Acustica 91, 409–420. Available online at: https://www.researchgate.net/profile/Adelbert_Bronkhorst/publication/229068125_Auditory_distance_perception_in_humans_A_summary_of_past_and_present_research/links/574c075c08ae74237f3ed74e/Auditory-distance-perception-in-humans-A-summary-of-past-and-present-research.pdf

[B59] Zarate J. M. (2013). The neural control of singing. Front. Hum. Neurosci. 7:237. 10.3389/fnhum.2013.00237 23761746 PMC3669747

[B60] Zatorre R. J. Baum S. R. (2012). Musical melody and speech intonation: singing a different tune. PLoS Biol. 10:5. 10.1371/journal.pbio.1001372 22859909 PMC3409119

