# Supplementary material for: Song Is More Memorable Than Speech Prosody: Discrete Pitches Aid Auditory Working Memory
Source: Front Psychol. 2020 Dec 10;11:586723. doi: 10.3389/fpsyg.2020.586723 (PMC7758421; doi:10.3389/fpsyg.2020.586723)
Supplement: Supplementary file 1 [file Data_Sheet_1.PDF]

Supplementary Material

Supplementary Information

The software package Praat (Version 6.0.36, Boersma & Weenink, 2017) was used to create the stimuli. The following parameters were used to isolate the pitch trajectories from the Mandarin phrases: function "To Pitch (ac)", parameters: time bin = 0.01; pitch floor = 40.0; maximum number of candidates = 15; accurate estimate = "yes"; silence threshold = 0.03; voicing threshold = 0.25; octave cost = 0.01; octave jump cost = 0.9; voiced-unvoiced cost = 0.9; pitch ceiling = 400.

Supplementary Figures and Tables

Supplementary Table 1: Characteristics of last syllables of speech contours that had pitch samples below 80 Hz. A: number of speech stimuli which had pitch samples <80 Hz/total number of speech stimuli. B: Frequency of lowest pitch sample across all of the concerned speech stimuli (Hz). C: Median of the set of lowest pitch samples of each concerned speech stimulus (Hz). D: Number of concerned speech stimuli of which the mean of the syllables containing lowest pitch samples was below 80 Hz. E: Lowest of all mean pitches of syllables containing lowest pitch samples. F: Median of all mean pitches of syllables containing lowest pitch samples.

Study	A: # <80Hz /# total	B: lowest pitch sample	C: median lowest pitch samples	D: # mean<80Hz	E: lowest mean pitch	F: median lowest mean pitches
1	37/94	65.66	73.35	24	72.37	78.92
2	30/76	65.77	73.42	22	72.37	79.41
3	17/45	65.66	73.35	8	76.64	78.92

Supplementary Table 2: Stability of GLMM in study 1, with minimum and maximum fixed effects parameter estimates after exclusion of levels of random effects one at a time, as well as the original model estimates.

Term	original	min	max
Intercept	-2.678	-2.833	-2.577
Stimulus State diff	2.860	2.760	2.985
Intermediate	0.197	0.097	0.286
Song	0.264	0.198	0.395
z.Musicality	0.295	0.114	0.446
Stimulus State diff: Intermediate	0.593	0.497	0.686
Stimulus State diff: Song	0.397	0.270	0.518
Stimulus State diff:z.Musicality	0.016	-0.101	0.149
Intermediate:z.Musicality	-0.309	-0.460	-0.235
Song:z.Musicality	-0.086	-0.157	0.058
Stimulus State diff: Intermediate:z.Musicality	0.616	0.510	0.686
Stimulus State diff:Song:z.Musicality	-0.010	-0.158	0.168

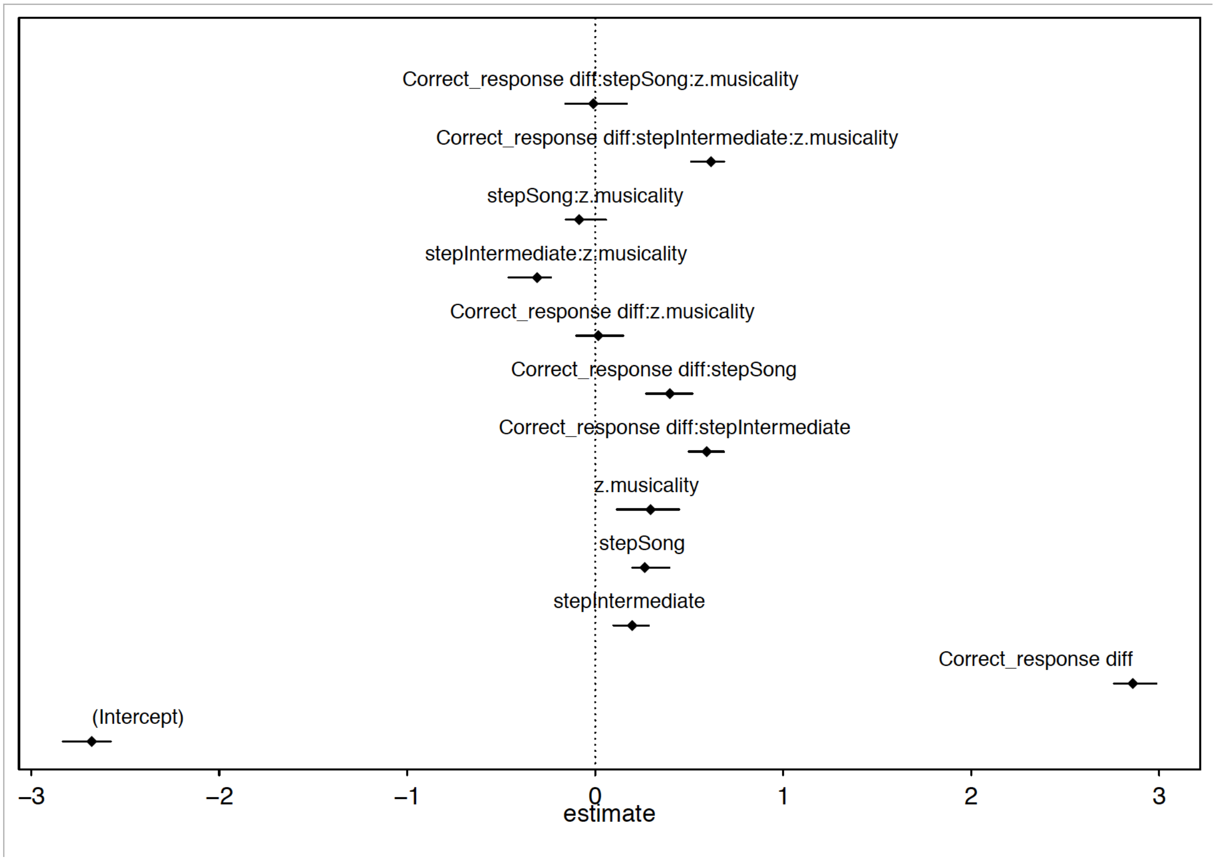

Supplementary Figure 1: Stability of GLMM in study 1, with range of fixed effects parameter estimates (straight lines) after exclusion of levels of random effects one at a time, as well as the original model estimates (diamond shapes).

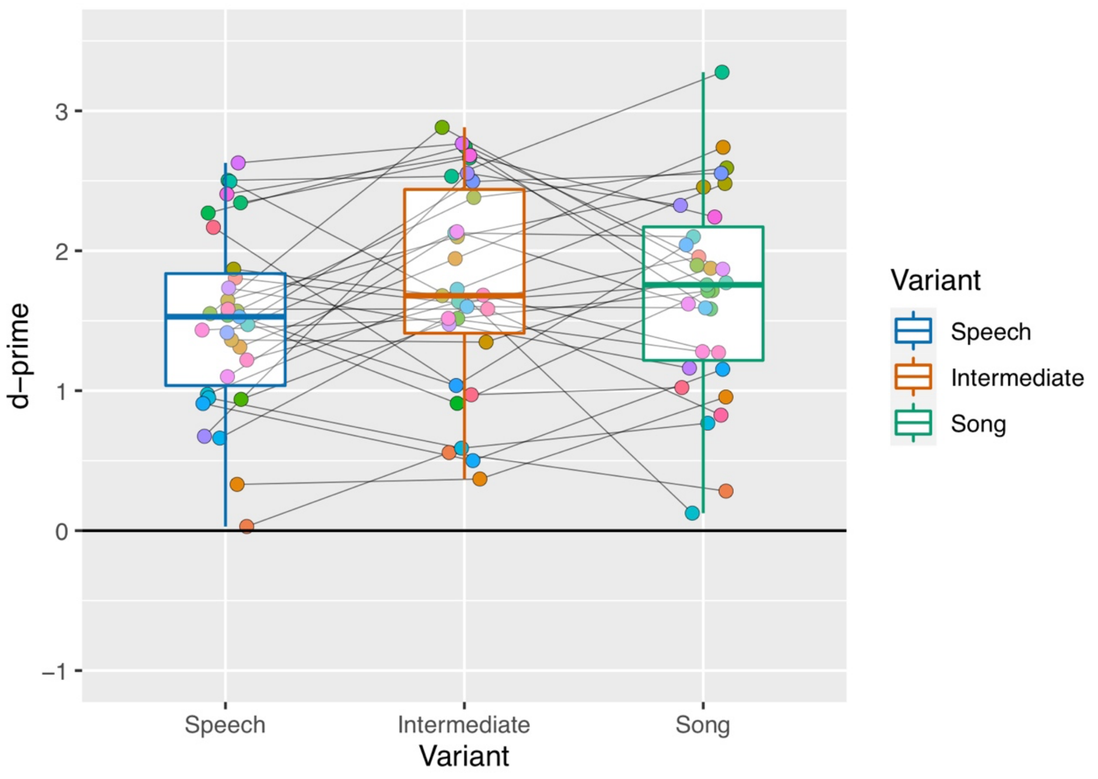

Supplementary Figure 2: d-primes as function of stimulus Variant in study 1. Grey lines connect values of individual, differently coloured participants. Correction for hit rates and false alarm rates of 0 and 1: +/- 1/(2N). N = 31.

Supplementary Table 3: Uncorrected hit rates and false alarm rates for participants and stimulus variants in study 1.

	Speech		Intermediate		Song	
Participant	Hit rate	False Alarm rate	Hit rate	False Alarm rate	Hit rate	False Alarm rate
1	0.700	0.100	0.818	0.238	0.750	0.100
2	0.304	0.294	0.611	0.391	0.474	0.364
3	0.381	0.263	0.526	0.381	0.700	0.333
4	0.524	0.105	0.600	0.045	0.684	0.000
5	0.409	0.056	0.588	0.130	0.714	0.095
6	0.500	0.050	0.864	0.158	0.778	0.045
7	0.609	0.056	0.600	0.077	0.591	0.000
8	0.636	0.111	0.762	0.048	0.882	0.080
9	0.444	0.045	0.875	0.042	0.654	0.067
10	0.636	0.278	0.789	0.238	0.842	0.238
11	0.478	0.056	0.737	0.391	0.722	0.130
12	0.813	0.083	0.947	0.130	0.840	0.278
13	0.750	0.048	0.929	0.115	0.808	0.188
14	0.826	0.059	0.800	0.045	0.941	0.043
15	0.600	0.000	0.619	0.091	0.316	0.000
16	0.519	0.077	0.647	0.040	0.750	0.077
17	0.526	0.182	0.682	0.105	0.316	0.273
18	0.381	0.105	0.500	0.278	0.471	0.200
19	0.800	0.571	0.895	0.364	0.857	0.300
20	0.500	0.182	0.250	0.120	0.636	0.211
21	0.238	0.000	0.250	0.043	0.421	0.000
22	0.400	0.048	0.789	0.045	0.619	0.000
23	0.750	0.500	1.000	0.381	1.000	0.471
24	0.545	0.053	0.556	0.091	0.600	0.182
25	0.647	0.000	0.700	0.000	0.609	0.056
26	0.125	0.000	0.455	0.000	0.500	0.053
27	0.565	0.000	0.667	0.000	0.500	0.000
28	0.529	0.087	0.762	0.211	0.727	0.250
29	0.444	0.087	0.667	0.105	0.667	0.200
30	0.588	0.087	0.722	0.160	0.560	0.250
31	0.471	0.000	0.450	0.136	0.435	0.118

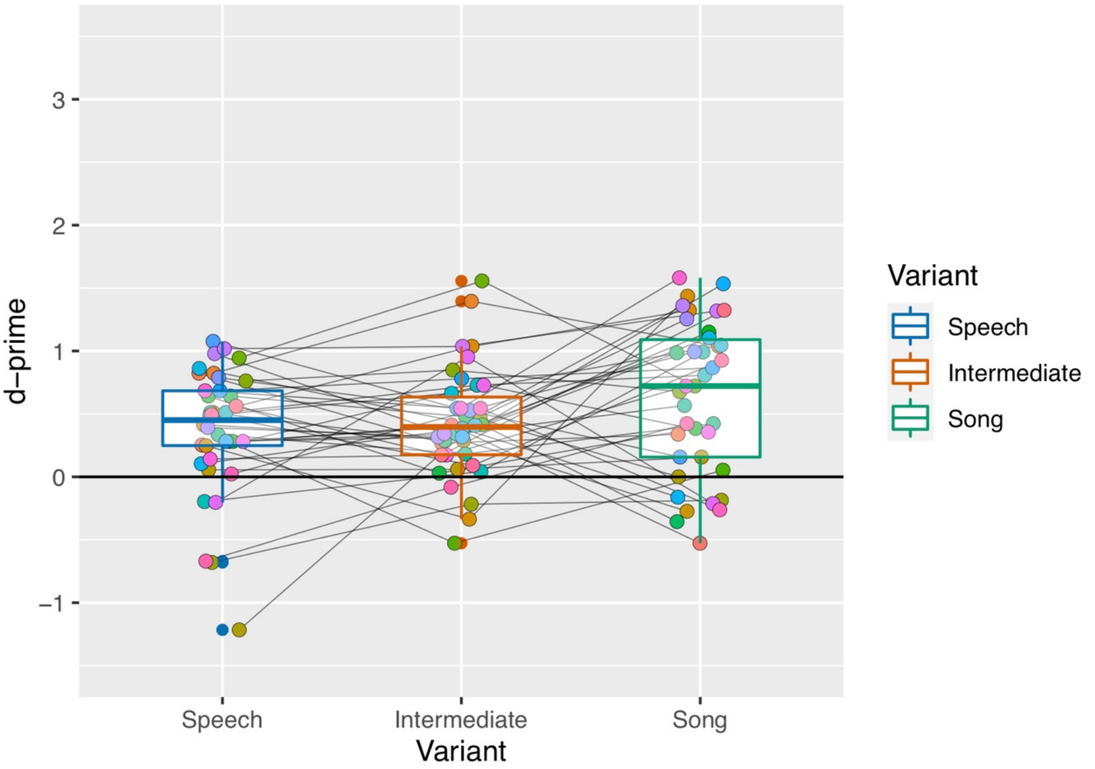

Supplementary Figure 3: d-primes as function of stimulus Variant in study 2. Grey lines connect values of individual participants. Correction for hit rates and false alarm rates of 0 and 1: +/- 1/(2N). N = 38.

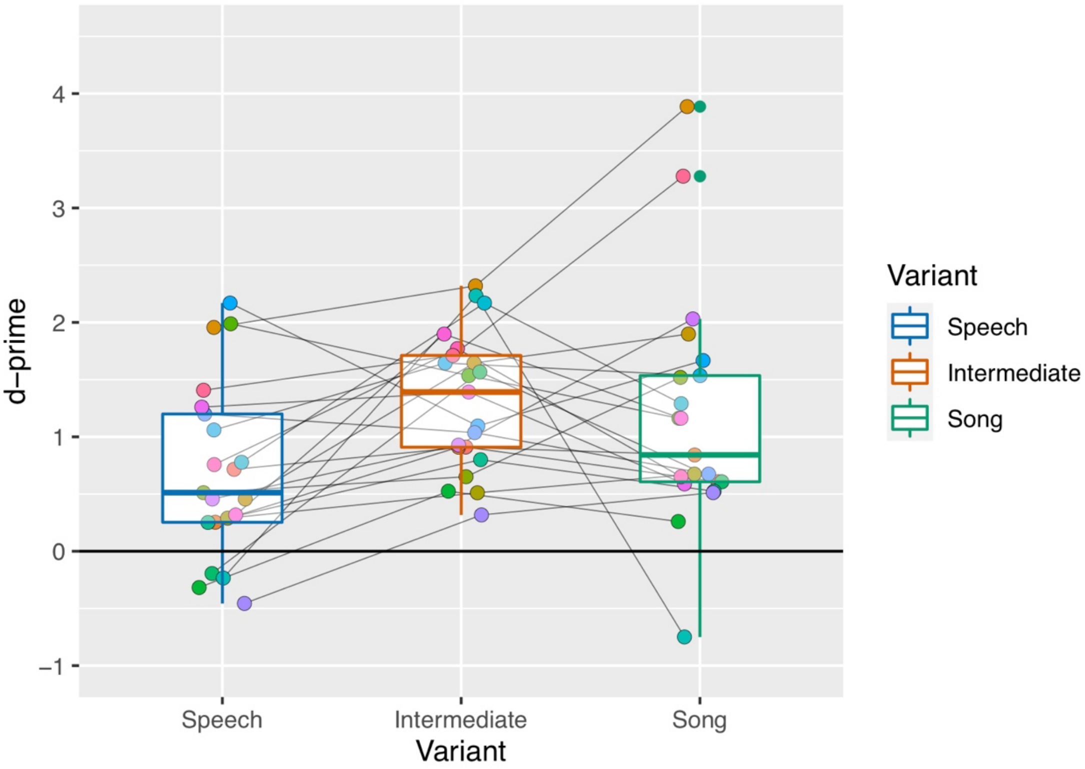

Supplementary Figure 4: d-primes as function of stimulus Variant in study 3. Grey lines connect values of individual participants. Correction for hit rates and false alarm rates of 0 and 1: +/- 1/(2N). N = 21.

Supplementary Table 4: Stability of GLMM in study 2, with minimum and maximum fixed effects parameter estimates after exclusion of levels of random effects one at a time, as well as the original model estimates.

Term	original	min	max
Intercept	-0.980	-1.064	-0.932
Stimulus State diff	0.550	0.448	0.623
Intermediate	-0.150	-0.230	-0.089
Song	-0.225	-0.280	-0.131
z.Musicality	-0.063	-0.169	-0.031
Stimulus State diff: Intermediate	0.044	-0.068	0.146
Stimulus State diff: Song	0.521	0.418	0.691
Stimulus State diff:z.Musicality	0.118	0.034	0.176
Intermediate:z.Musicality	0.012	-0.065	0.104
Song:z.Musicality	0.204	0.149	0.287
Stimulus State diff: Intermediate:z.Musicality	0.125	0.027	0.193
Stimulus State diff:Song:z.Musicality	0.032	-0.105	0.113

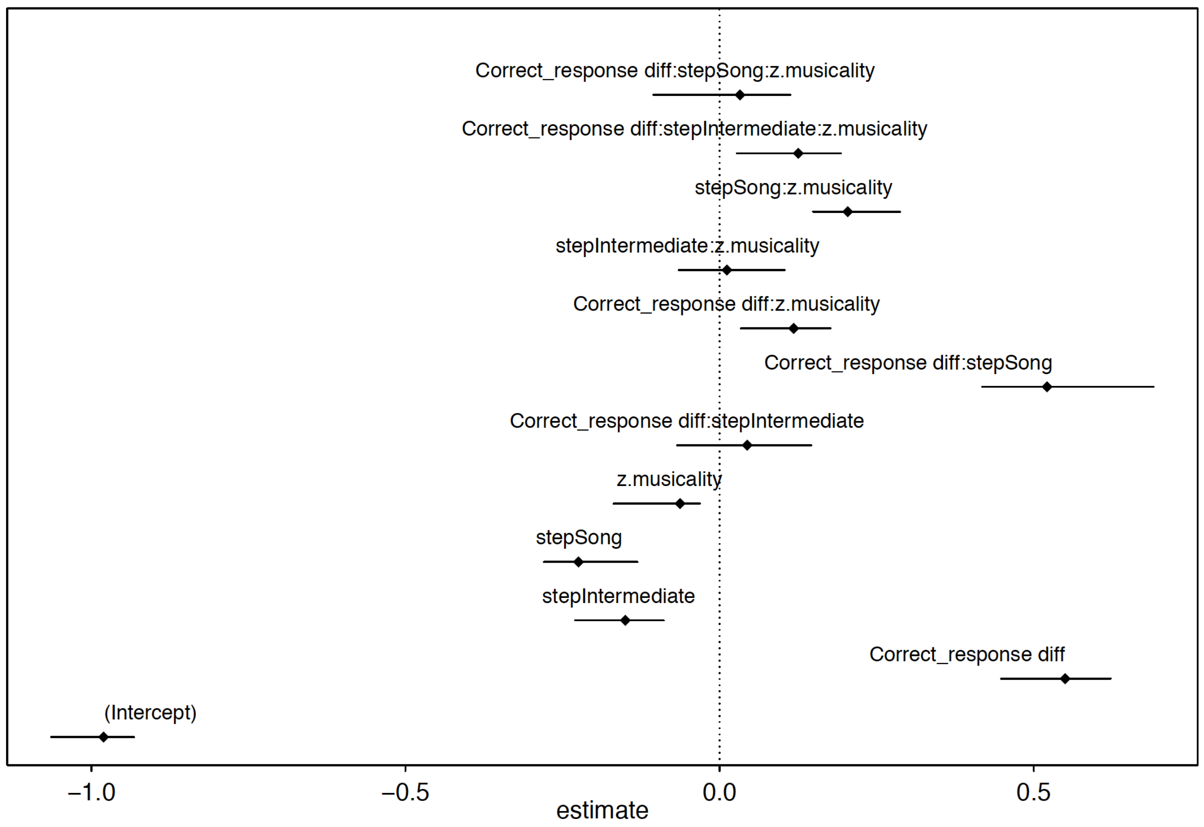

Supplementary Figure 5: Stability of GLMM in study 2, with range of fixed effects parameter estimates (straight lines) after exclusion of levels of random effects one at a time, as well as the original model estimates (diamond shapes).

Supplementary Table 5: Uncorrected hit rates and false alarm rates for participants and stimulus variants in study 2.

	Speech		Intermediate		Song	
Participant	Hit rate	False Alarm rate	Hit rate	False Alarm rate	Hit rate	False Alarm rate
1	0.600	0.500	0.333	0.200	0.462	0.667
2	0.267	0.074	0.143	0.091	0.208	0.125
3	0.267	0.074	0.524	0.091	0.708	0.313
4	0.400	0.222	0.476	0.136	0.417	0.063
5	0.467	0.370	0.286	0.409	0.708	0.188
6	0.533	0.370	0.476	0.364	0.333	0.438
7	0.467	0.444	0.524	0.500	0.500	0.438
8	0.000	0.148	0.190	0.091	0.250	0.250
9	0.133	0.333	0.286	0.364	0.250	0.313
10	0.667	0.370	0.476	0.182	0.417	0.188
11	0.200	0.037	0.238	0.000	0.208	0.063
12	0.400	0.296	0.190	0.364	0.583	0.563
13	0.400	0.296	0.476	0.318	0.500	0.125
14	0.400	0.185	0.143	0.136	0.458	0.313
15	0.400	0.185	0.333	0.182	0.250	0.375
16	0.400	0.222	0.476	0.364	0.292	0.063
17	0.333	0.222	0.286	0.227	0.542	0.375
18	0.400	0.296	0.286	0.182	0.167	0.063
19	0.333	0.407	0.333	0.318	0.542	0.188
20	0.400	0.222	0.429	0.182	0.625	0.313
21	0.600	0.407	0.524	0.273	0.458	0.125
22	0.667	0.333	0.524	0.364	0.667	0.250
23	0.333	0.296	0.238	0.136	0.375	0.438
24	0.400	0.296	0.333	0.227	0.500	0.063
25	0.600	0.333	0.667	0.364	0.708	0.375
26	0.667	0.259	0.476	0.273	0.500	0.438
27	0.600	0.296	0.571	0.364	0.750	0.375
28	0.400	0.259	0.333	0.227	0.542	0.125
29	0.533	0.185	0.286	0.182	0.583	0.125
30	0.600	0.222	0.476	0.136	0.667	0.188
31	0.800	0.852	0.857	0.545	0.750	0.813
32	0.400	0.296	0.429	0.182	0.375	0.250
33	0.267	0.222	0.333	0.273	0.208	0.063
34	0.600	0.333	0.476	0.273	0.667	0.125
35	0.267	0.259	0.476	0.273	0.458	0.563
36	0.267	0.519	0.333	0.364	0.542	0.375
37	0.364	0.200	0.154	0.133	0.133	0.000
38	0.467	0.259	0.333	0.273	0.417	0.063

Supplementary Table 6: Stability of GLMM in study 3, with minimum and maximum fixed effects parameter estimates after exclusion of levels of random effects one at a time, as well as the original model estimates.

Term	original	min	max
Intercept	-1.478	-1.566	-1.392
Stimulus State diff	1.156	1.042	1.253
Intermediate	-0.137	-0.238	-0.070
Song	-0.169	-0.256	-0.093
z.Musicality	-0.218	-0.358	-0.172
Stimulus State diff: Intermediate	0.867	0.739	1.023
Stimulus State diff: Song	0.832	0.749	0.959
Stimulus State diff:z.Musicality	0.456	0.350	0.607
Intermediate:z.Musicality	-0.030	-0.121	0.049
Song:z.Musicality	-0.175	-0.303	0.060
Stimulus State diff: Intermediate:z.Musicality	0.054	-0.024	0.181
Stimulus State diff:Song:z.Musicality	0.297	0.202	0.399

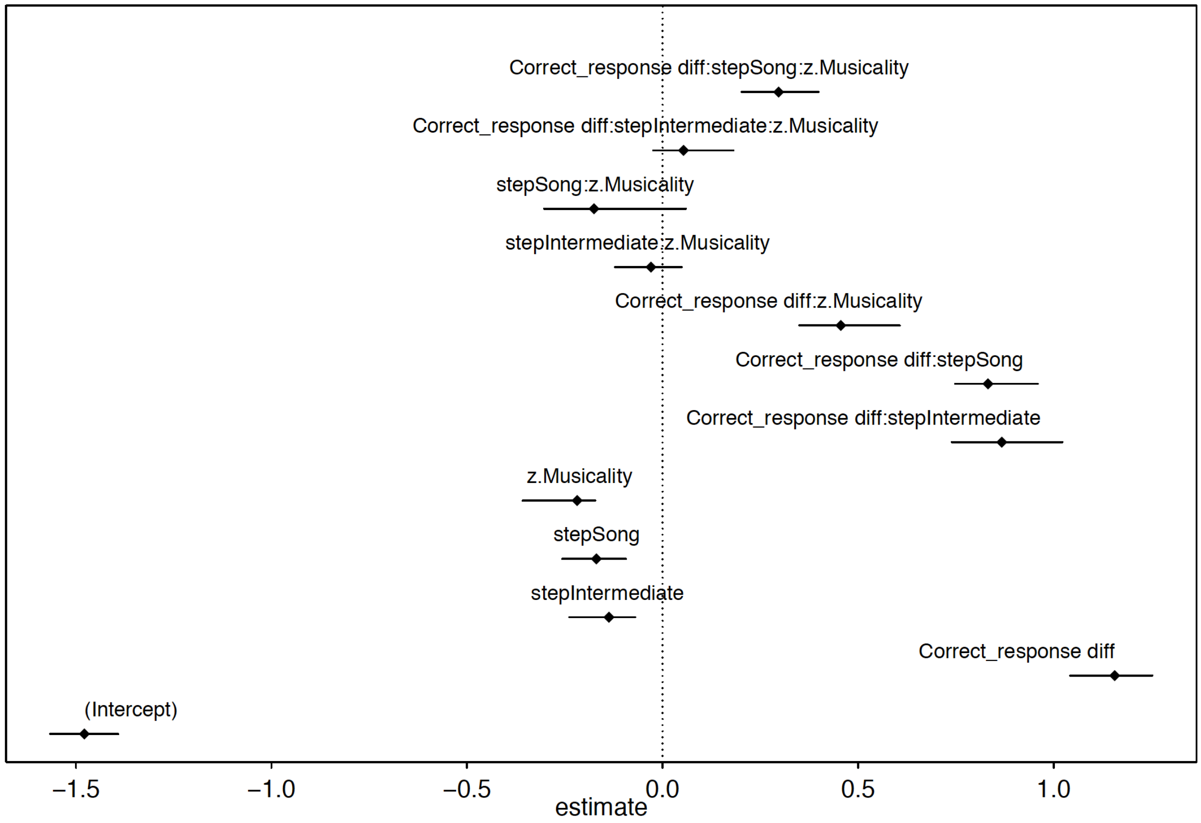

Supplementary Figure 6: Stability of GLMM in study 3, with range of fixed effects parameter estimates (straight lines) after exclusion of levels of random effects one at a time, as well as the original model estimates (diamond shapes).

Supplementary Table 7: Uncorrected hit rates and false alarm rates for participants and stimulus variants in study 3.

	Speech		Intermediate		Song	
Participant	Hit rate	False Alarm rate	Hit rate	False Alarm rate	Hit rate	False Alarm rate
1	0.450	0.200	0.450	0.150	0.250	0.100
2	0.500	0.400	0.700	0.350	0.500	0.200
3	0.750	0.100	0.750	0.050	0.950	0.000
4	0.350	0.200	0.500	0.050	0.600	0.050
5	0.350	0.250	0.550	0.350	0.500	0.250
6	0.300	0.150	0.350	0.150	0.450	0.050
7	0.400	0.000	0.600	0.100	0.550	0.150
8	0.200	0.300	0.500	0.300	0.650	0.550
9	0.150	0.200	0.250	0.000	0.250	0.100
10	0.550	0.450	0.550	0.250	0.500	0.300
11	0.182	0.250	0.667	0.000	0.375	0.667
12	0.600	0.300	0.950	0.300	0.850	0.400
13	0.750	0.350	0.950	0.500	0.900	0.400
14	0.700	0.050	0.600	0.200	0.650	0.100
15	0.750	0.300	0.850	0.500	0.750	0.500
16	0.200	0.350	0.300	0.200	0.300	0.150
17	0.350	0.200	0.600	0.250	0.650	0.050
18	0.350	0.050	0.400	0.050	0.400	0.200
19	0.300	0.200	0.600	0.050	0.550	0.150
20	0.300	0.100	0.550	0.050	0.350	0.150
21	0.550	0.100	0.750	0.150	0.850	0.000

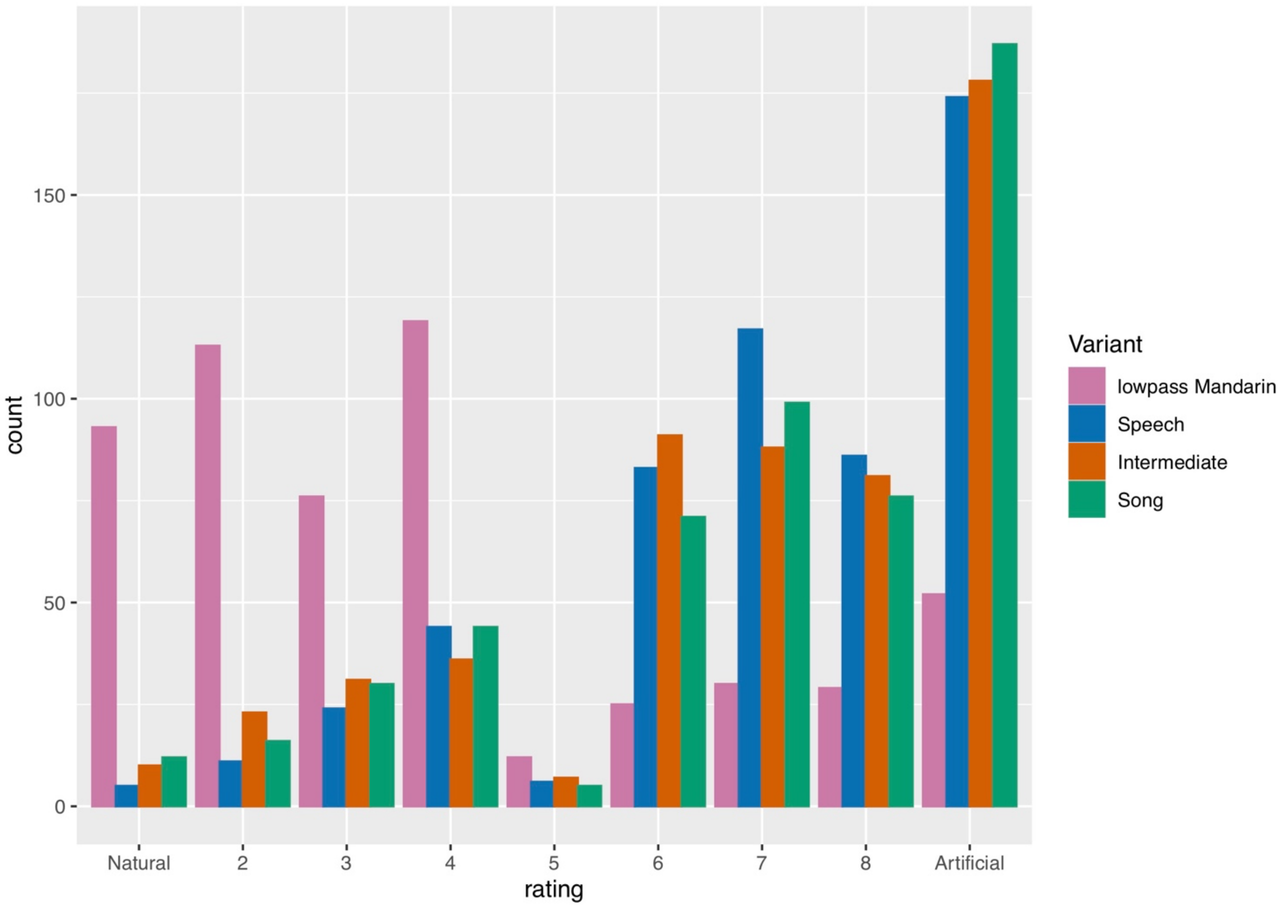

Supplementary Figure 7: Rating counts obtained in the post-hoc rating study on the question how natural or artificial the stimuli sounded. Stimuli presented were those used in studies 1, 2 and 3 (Song, Speech and Intermediate) as well as the original, lowpass-filtered Mandarin Chinese phrases. Rating was done on a 9-point Likert scale following the question "How does it sound like?". N = 56.

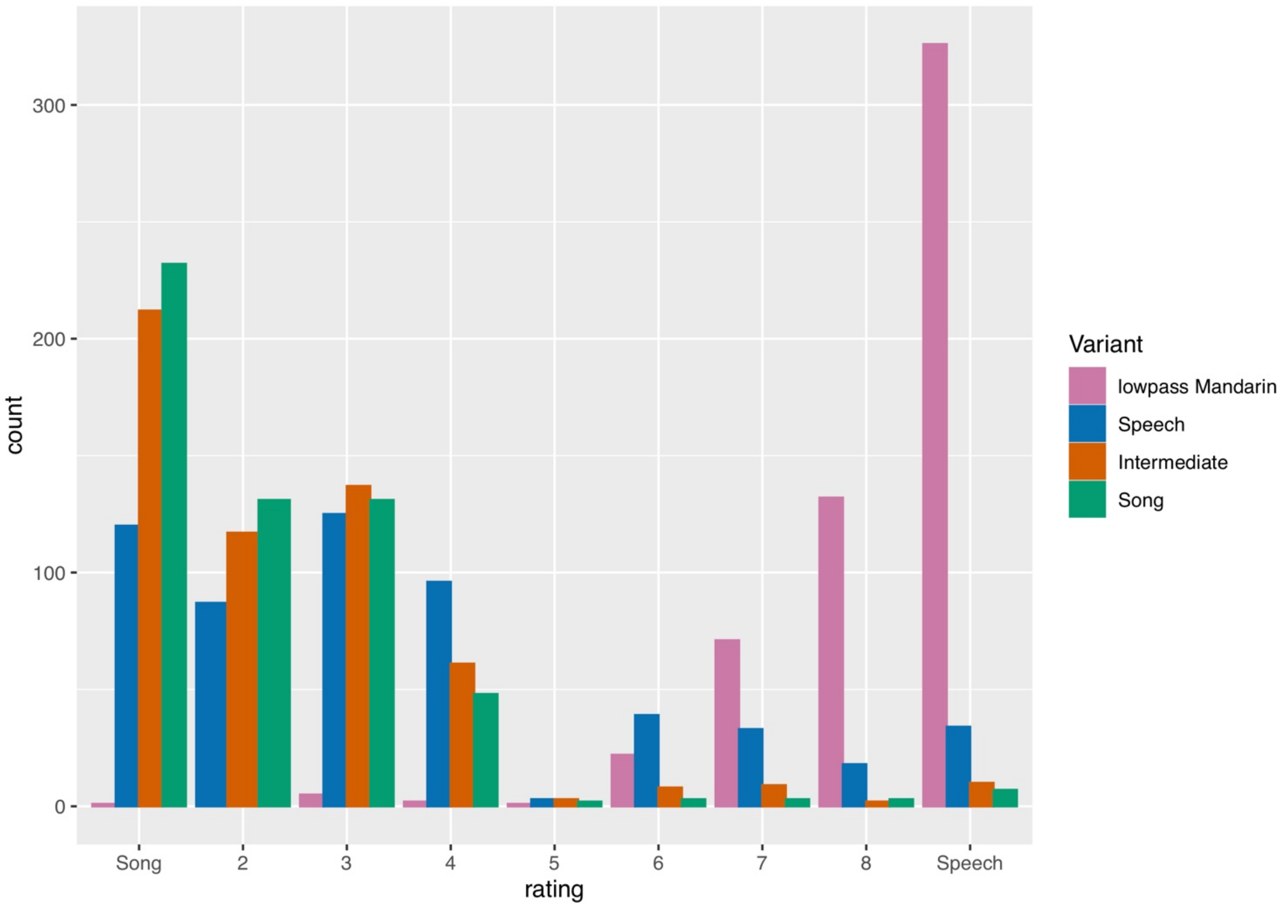

Supplementary Figure 8: Rating counts obtained in the post-hoc rating study on the question how song-like or speech-like the stimuli sounded. Stimuli presented were those used in studies 1, 2 and 3 (Song, Speech and Intermediate) as well as the original, lowpass-filtered Mandarin Chinese phrases. Rating was done on a 9-point Likert scale following the question "How does it sound like?". N = 56.

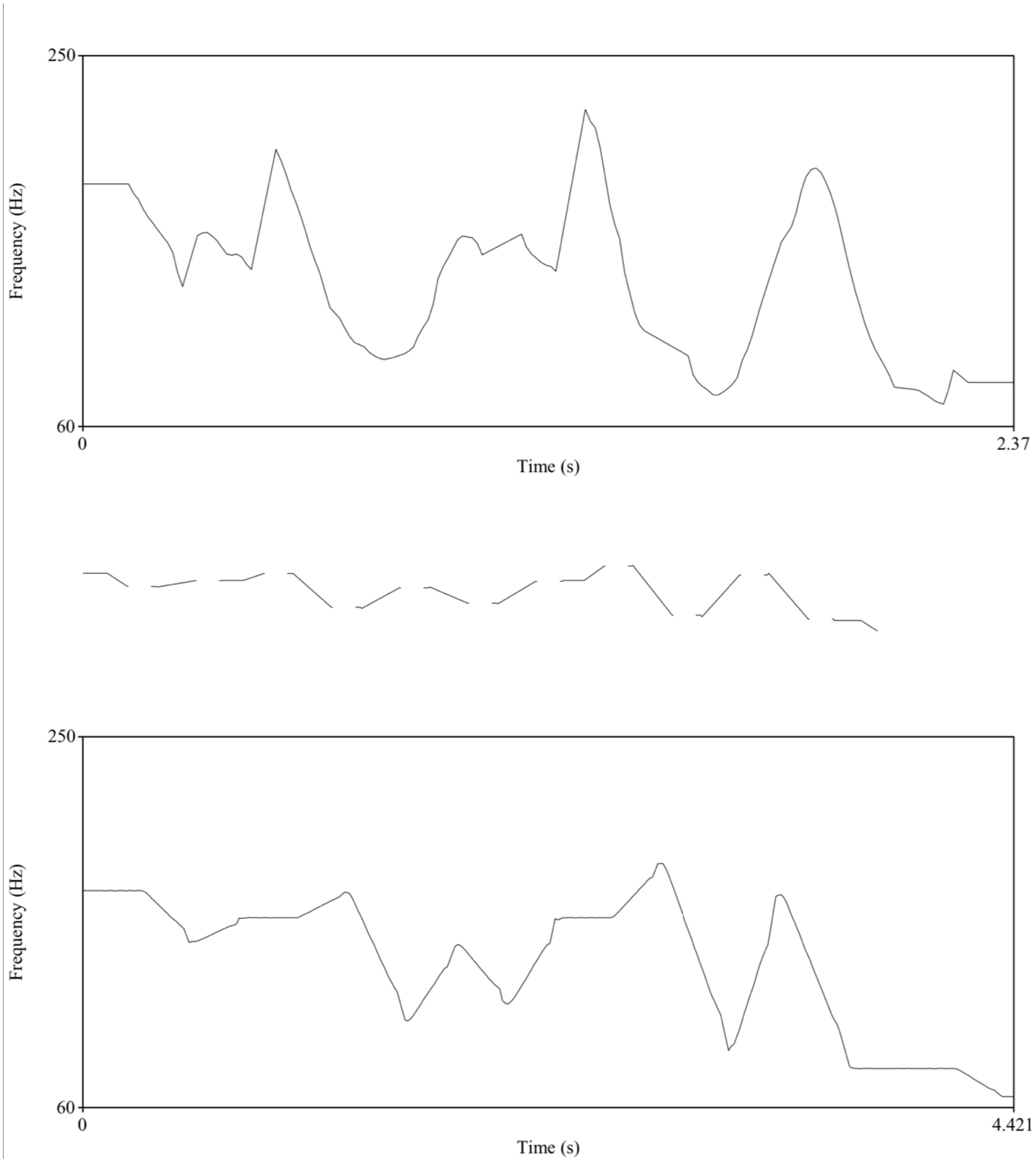

Supplementary Figure 9: Top: pitch contour of original Mandarin phrase that provided the basis for stimulus Nr. 35. Middle: single pitch contour chunks (not true to time scale) extracted from original, shifted to Bohlen-Pierce scale and occasionally lengthened. Bottom: all of these pitch contour chunks concatenated. For the actual stimulus, chunks 1 to 6 (study 2: 1 to 9) and the last chunk had been concatenated. Plots were created with Praat (Version 6.0.36, Boersma & Weenink, 2017).

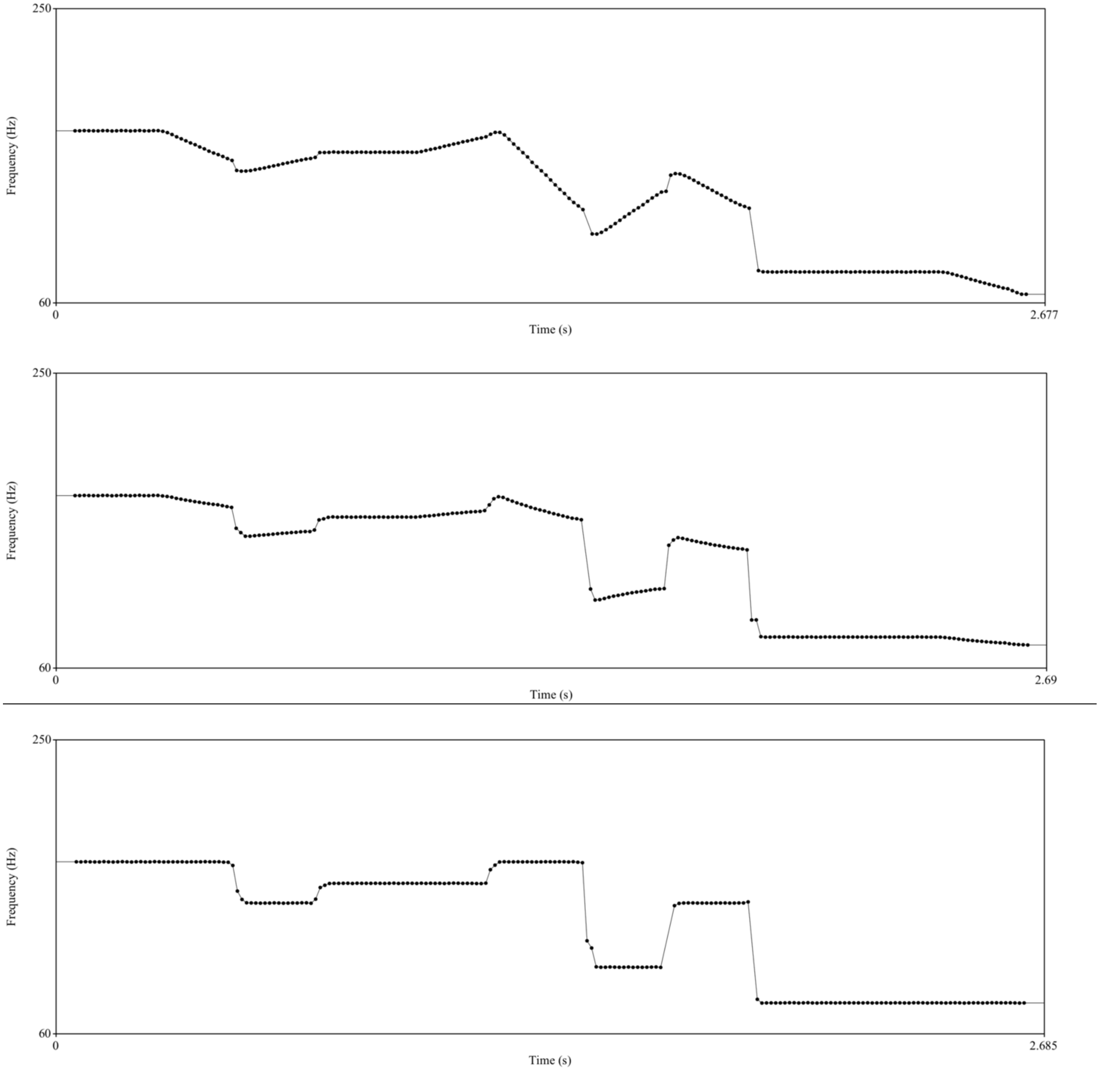

Supplementary Figure 10: The three versions of stimulus Nr. 35. Top: Speech prosody version. Middle: Intermediate version. Bottom: Song version. Plots were created with Praat (Version 6.0.36, Boersma & Weenink, 2017).

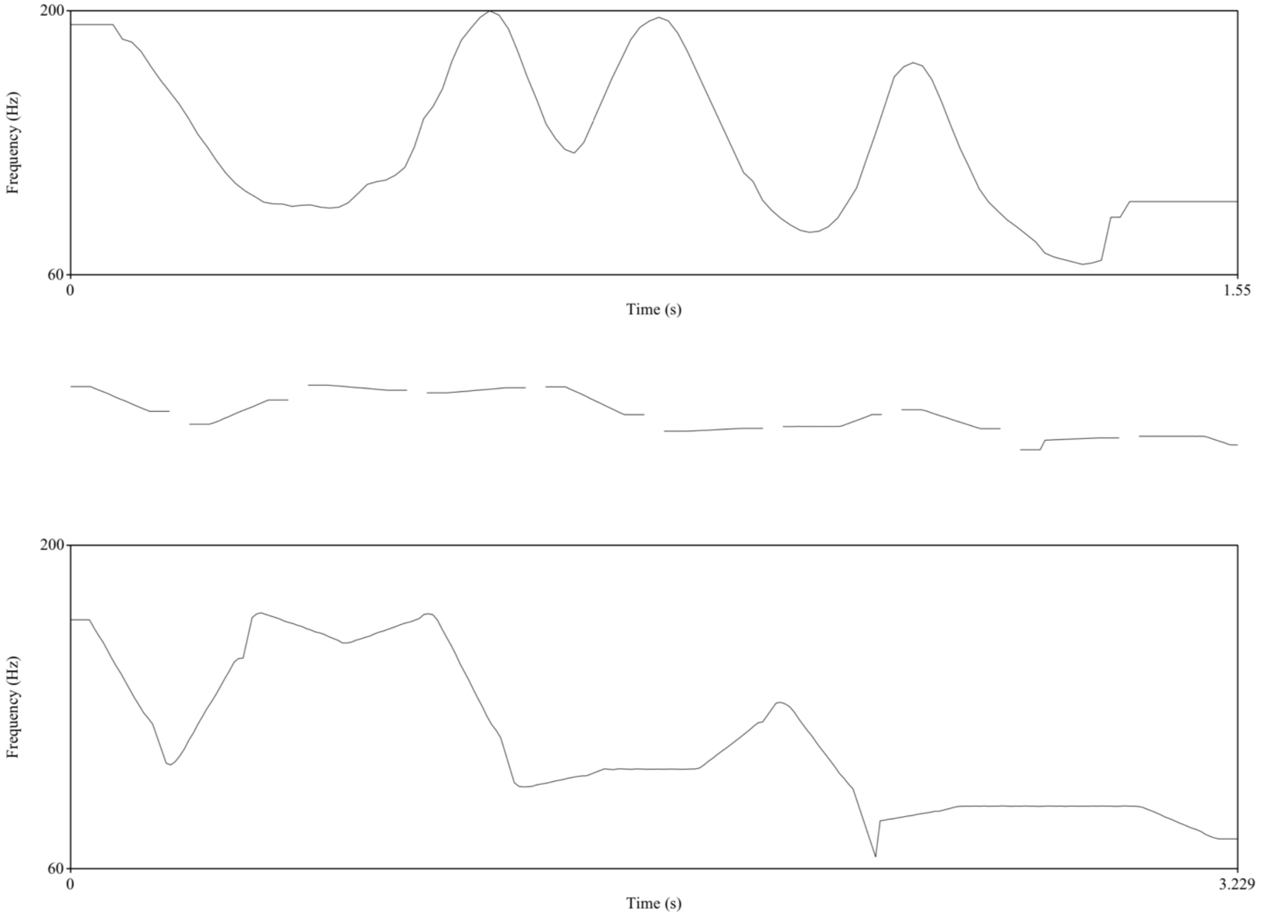

Supplementary Figure 11: Top: pitch contour of original Mandarin phrase that provided the basis for stimulus Nr. 182. Middle: single pitch contour chunks (not true to time scale) extracted from original, shifted to Bohlen-Pierce scale and occasionally lengthened. Bottom: all of these pitch contour chunks concatenated. For the actual stimulus, chunks 1 to 6 (study 2: 1 to 9) and the last chunk had been concatenated. Plots were created with Praat (Version 6.0.36, Boersma & Weenink, 2017).

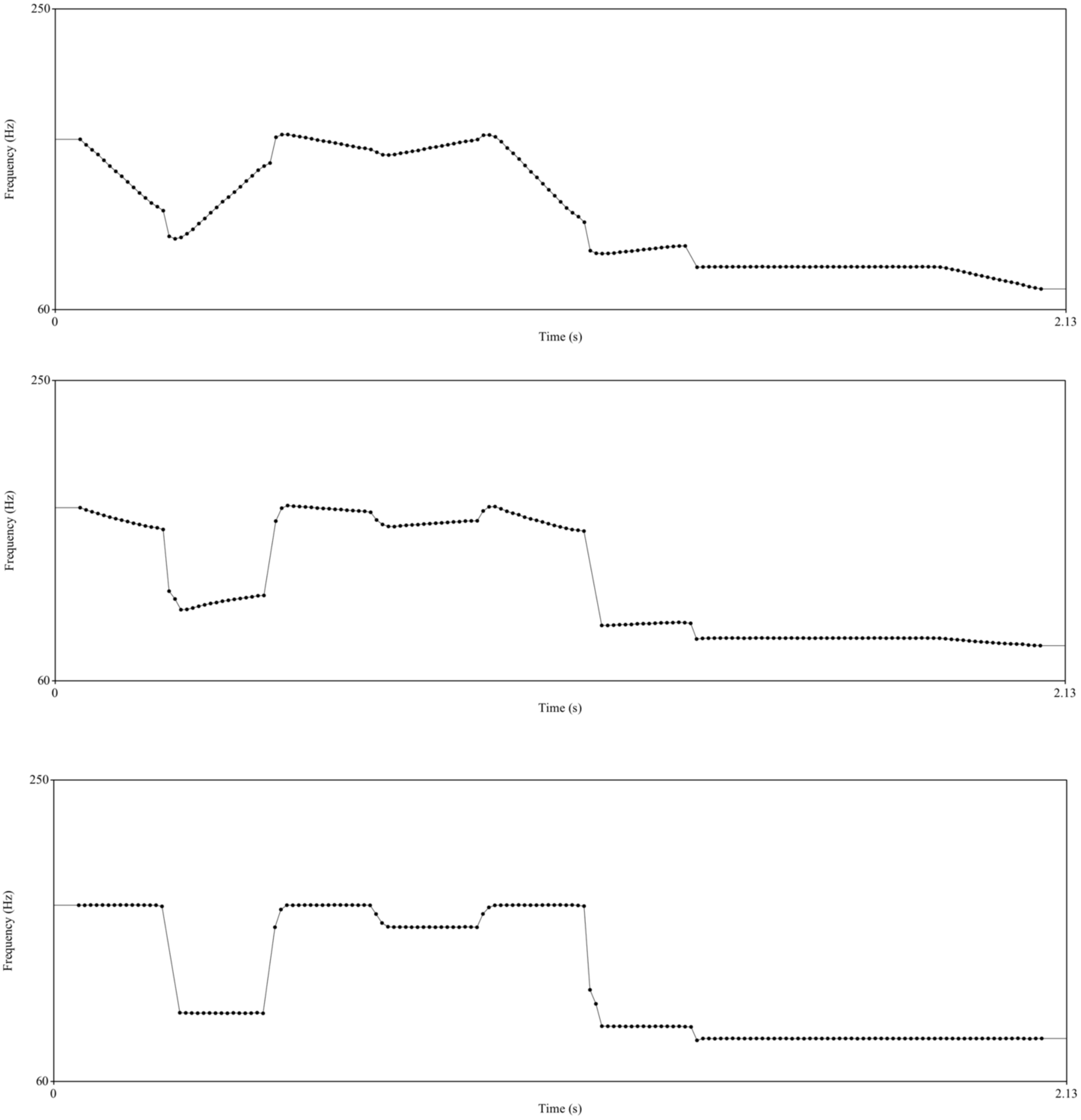

Supplementary Figure 12: The three versions of stimulus Nr. 182. Top: Speech prosody version. Middle: Intermediate version. Bottom: Song version. Plots were created with Praat (Version 6.0.36, Boersma & Weenink, 2017).

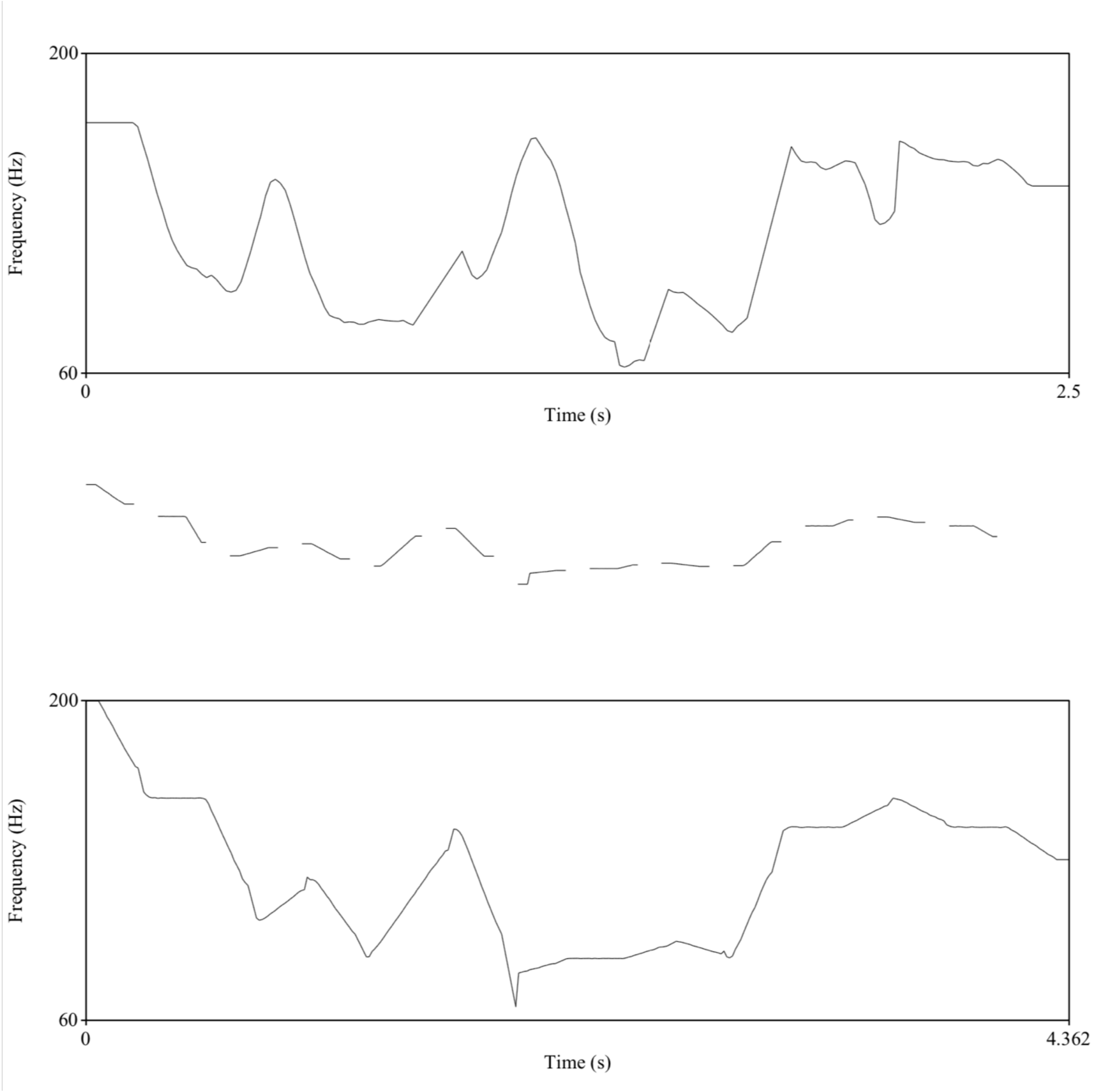

Supplementary Figure 13: Top: pitch contour of original Mandarin phrase that provided the basis for stimulus Nr. 10. Middle: single pitch contour chunks (not true to time scale) extracted from original, shifted to Bohlen-Pierce scale and occasionally lengthened. Bottom: all of these pitch contour chunks concatenated. For the actual stimulus, chunks 1 to 6 (study 2: 1 to 9) and the last chunk had been concatenated. Plots were created with Praat (Version 6.0.36, Boersma & Weenink, 2017).

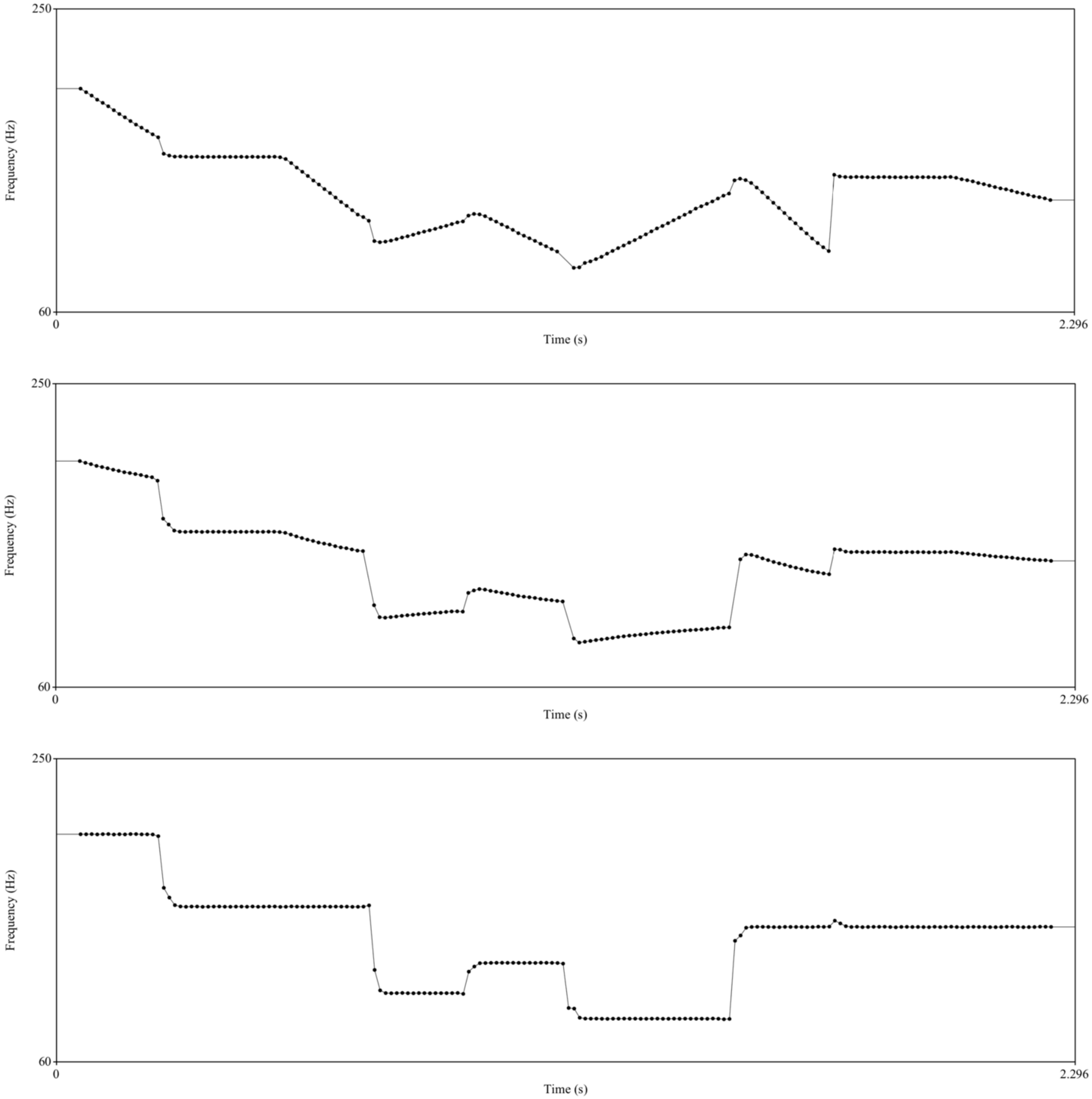

Supplementary Figure 14: The three versions of stimulus Nr. 10. Top: Speech prosody version. Middle: Intermediate version. Bottom: Song version. Plots were created with Praat (Version 6.0.36, Boersma & Weenink, 2017).

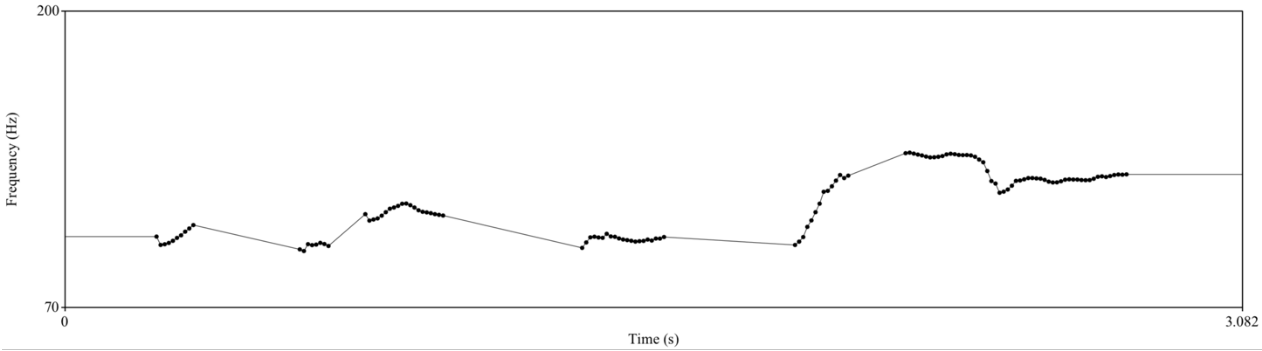

Supplementary Figure 15: Example for natural song pitch contour of "Happy Birthday", sung by the same male who provided the basis for the timbre of our stimuli.

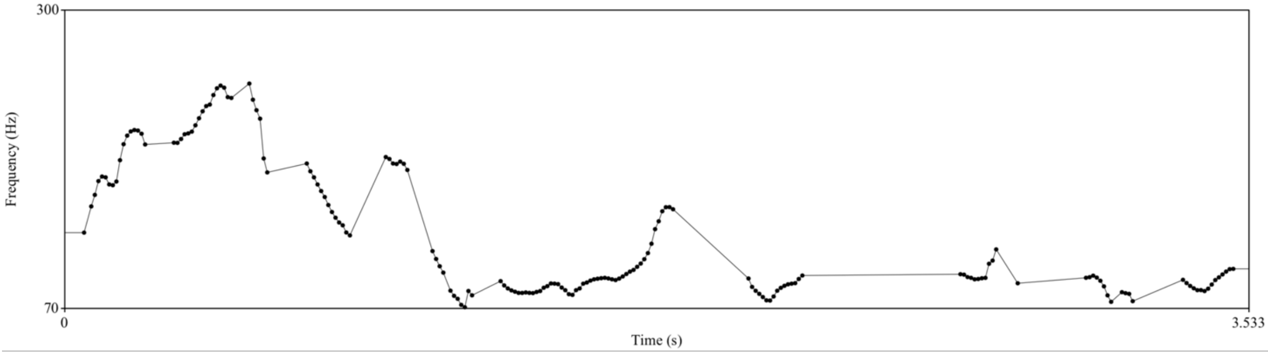

Supplementary Figure 16: Example for natural speech pitch contour of an English sentence, spoken by the same male who provided the basis for the timbre of our stimuli.

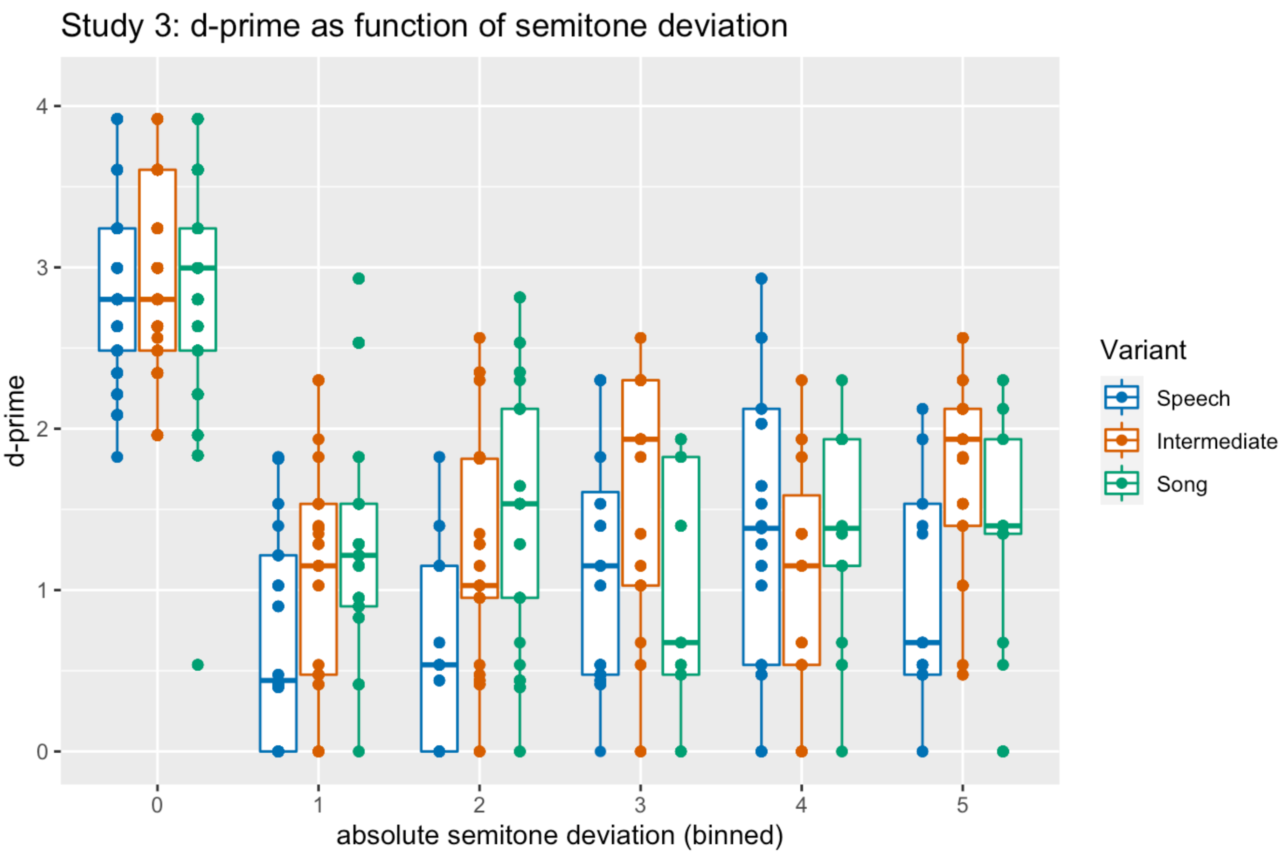

Supplementary Figure 17: d-primes as function of absolute semitone deviation in study 3. Semitone deviations were measured in Praat as real numbers, but for better visualization, binned semitone deviations are plotted here. For comparability in this plot only, d-primes for 0 deviation (standards) were calculated based on a different definition of participants' responses: hits were defined as detection of a standard when a standard was presented (false alarms, correct rejections and misses accordingly). Hit rates and False Alarm rates of 0/1 were corrected by adding/subtracting 1/2n (n being the number of trials) (Macmillan & Creelman, 2005)
